# Unlocking the Potential of Pyrrole: Recent Advances in New Pyrrole-Containing Compounds with Antibacterial Potential

**DOI:** 10.3390/ijms252312873

**Published:** 2024-11-29

**Authors:** Aura Rusu, Octavia-Laura Oancea, Corneliu Tanase, Livia Uncu

**Affiliations:** 1 Pharmaceutical and Therapeutic Chemistry Department, Faculty of Pharmacy, George Emil Palade University of Medicine, Pharmacy, Science and Technology of Targu Mures, 540142 Targu Mures, Romania; aura.rusu@umfst.ro; 2 Organic Chemistry Department, Faculty of Pharmacy, George Emil Palade University of Medicine, Pharmacy, Science and Technology of Targu Mures, 540142 Targu Mures, Romania; octavia.moldovan@umfst.ro; 3 Pharmaceutical Botany Department, Faculty of Pharmacy, George Emil Palade University of Medicine, Pharmacy, Science and Technology of Targu Mures, 540142 Targu Mures, Romania; 4 Scientific Center for Drug Research, Pharmaceutical and Toxicological Chemistry Department, “Nicolae Testemitanu” State University of Medicine and Pharmacy, 165 Bd. Stefan Cel Mare si Sfant, MD-2004 Chisinau, Moldova; livia.uncu@usmf.md

**Keywords:** pyrrole, pyrrole derivatives, pyrrole-based compounds, substituted pyrroles, heterocycles, five-membered heterocycles, antibiotics, antibacterials, antibacterial activity, nitrogen heterocycles

## Abstract

Nitrogen heterocycles are valuable structural elements in the molecules of antibacterial drugs approved and used to treat bacterial infections. Pyrrole is a five-atom heterocycle found in many natural compounds with biological activity, including antibacterial activity. Numerous compounds are being develop based on the pyrrole heterocycle as new potential antibacterial drugs. Due to the phenomenon of antibacterial resistance, there is a continuous need to create new effective antibacterials. In the scientific literature, we have identified the most relevant studies that aim to develop new compounds, such as pyrrole derivatives, that are proven to have antibacterial activity. Nature is an endless reservoir of inspiration for designing new compounds based on the structure of pyrrole heterocycles such as calcimycin, lynamycins, marinopyrroles, nargenicines, phallusialides, and others. However, many other synthetic compounds based on the pyrrole heterocycle have been developed and can be optimized in the future. The identified compounds were classified according to the type of chemical structure. The chemical structure–activity relationships, mechanisms of action, and antibacterial effectiveness of the most valuable compounds were highlighted. This review highlights scientific progress in designing new pyrrole-containing compounds and provides examples of lead compounds that can be successfully optimized further.

## 1. Introduction

### 1.1. The Role of Nitrogen Heterocycles in the Discovery of New Antibacterials

Heterocyclic compounds are essential in developing new drugs due to their physicochemical and biological properties. They interact with various receptors and enzymes in the body, leading to specific biological activities and therapeutic effects. These compounds can modulate factors such as potency, selectivity, lipophilicity, polarity, and aqueous solubility, ultimately improving drug candidates and their properties [1]. Medicinal chemists have exploited numerous heterocyclic compounds to create new antibacterials that efficiently treat bacterial infections. Five-membered nitrogen heterocycles, containing two or three heteroatoms, are critical structural components in several antibacterials successfully used to treat bacterial infections caused by various pathogens [2].

Heterocyclic ring structures are present in many substances, including hemoglobin, synthetic medicines, vitamins, hormones, alkaloids, and dyes. Nitrogen-containing heterocycles have been the subject of intensive research because of their numerous and essential uses in organic synthesis, agriculture, and biology [2]. Nitrogen heterocycles are valuable structural elements in the molecules of antibacterial drugs approved and used to treat bacterial infections. The chemical structures of FDA-approved antibiotics that contain one or more nitrogen heterocycles like pyrrolidine, tetrazole, 1,2,3-triazole, and pyrrole (one nitrogen atom) are summarized in Table 1; there, we present the FDA-approved drugs since 1980 (and covering more than four decades). 

Bacterial infection remains a common medical issue worldwide. Antibacterial drugs have saved many lives, proving effective weapons against bacterial infections since their discovery. However, antibacterial resistance now severely threatens human health, primarily due to the widespread use of antibacterial drugs [4]. Resistance arises from the various mechanisms that bacteria develop to evade antibiotic effects, including intrinsic and acquired resistance, genetic changes, and DNA transfer [5]. The ongoing battle against bacterial infections urgently requires new and effective antibacterial drugs. Unfortunately, despite this, pharmaceutical companies are becoming increasingly reluctant to finance the discovery and development of new antibacterials [6].

### 1.2. Aim of This Work 

Our recent work focused on the relationships between known antibacterials’ chemical structures containing five-membered heterocycles and their biological properties. This review article thoroughly examines the five-atom heterocycles used to design approved antibacterial drugs [7]. To continue the central idea of our work, we comprehensively set out to identify, organize, and analyze the new compounds under study, which include heterocycles with five atoms in their molecular structure, to potentially become antibacterial drugs. This paper focuses explicitly on the pyrrole heterocycle. Also, the study of chemical structure–activity relationships (SARs) of various series of compounds based on pyrrole and the mechanisms of action (where they are detailed) will be analyzed and discussed. The antibacterial effectiveness of the most valuable discovered compounds is underlined by the minimum inhibitory concentration (MIC) values or the diameter values of the inhibition zone. This review aims to highlight scientific progress in designing new antibacterials that contain the pyrrole heterocycle and provides examples of lead compounds that can be successfully optimized in future.

## 2. Short Description of the Pyrrole Heterocycle 

**Figure 1:**
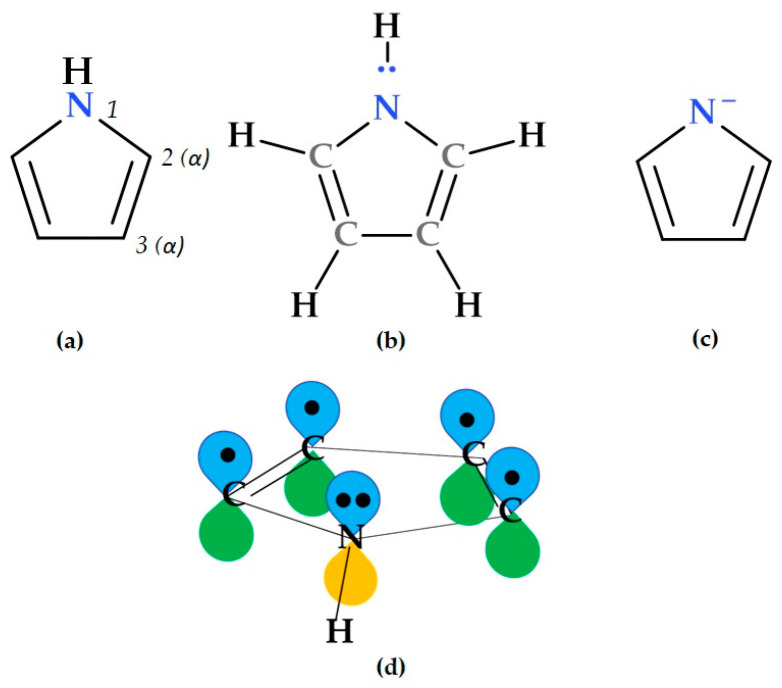
Molecular structure of pyrrole: (**a**) bond-line structure, (**b**) Lewis structure, (**c**) pyrrole anion, and (**d**) orbital structure of pyrrole with sp^2^ hybridized nitrogen (lone pair is part of the aromatic sextet) [8,9,11].

Pyrrole is a heterocyclic ring with five members associated with the formula C_4_H_5_N (Figure 1). All its atoms lie in the same plane, forming a nearly regular pentagon. The nitrogen atom in pyrrole is sp^2^ hybridized. Pyrrole is aromatic due to an aromatic sextet composed of six π electrons. Each carbon contributes one π electron, and the nitrogen contributes two from its lone pair. Pyrrole has an excess of π electrons, with electron density greater than 1 on each atom within the ring. Pyrrole is more aromatic than furan but less aromatic than thiophene (thiophene > pyrrole > furan) [8,9,10].

Pyrrol has the following physical properties: it is a colourless volatile liquid that becomes dark upon exposure to air and has a density of 0.967 g/cm^3^ and a boiling point of 129–131 °C. It has a dipole moment of 1.58 D, with the positive end near the nitrogen atom. Position 2 protonation generates the thermodynamically stable pyrrolium cation (C_4_H_6_N^+^). Substituting pyrrole with alkyl groups increases its basicity. Various methods exist for synthesizing pyrroles, including insertion reactions, ring contraction and ring expansion reactions, and catalytic and noncatalytic cyclization reactions. These approaches allow for the construction of diverse pyrrole derivatives [12]. 

Due to its lipophilic character (logP 0.75), pyrrole can pass across the blood–brain barrier, penetrate the lipid layers of cell membranes, and be rapidly absorbed through passive diffusion. Apart from these particular properties, pyrrole has a modest hydrophilicity that makes it possible to interact with receptors and to achieve rapid blood circulation, easy metabolization, and the accumulation in bacterial agents [13,14].

The pyrrole heterocycle can participate in numerous chemical reactions, such as electrophilic substitution reactions (at ring nitrogen or ring carbon), nitration, sulfonation, acylation, the Vilsmeier reaction, the Houben–Hoesch reaction, oxidation, etc. [12]. Research on pyrrole derivatives in heteroaromatic chemistry is essential. These derivatives participate in chemical reactions, undergo synthesis, and show biological activity. Interestingly, pyrrole is an essential component of many natural products and medicines [15].

## 3. Natural Compounds Containing Pyrrole Heterocycle

**Figure 2:**
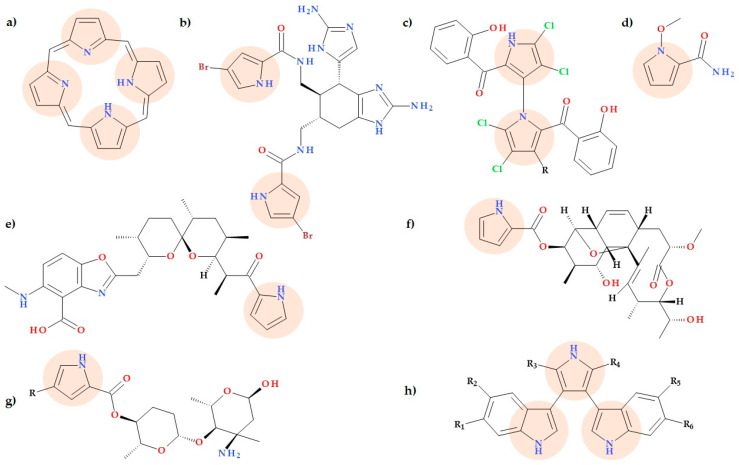
Molecular structure of some natural compounds containing pyrrole heterocycle(s): (**a**) Porphyrin, (**b**) Ageliferin, (**c**) Marinopyrole A and B (R = H or Br), (**d**) 1-Methoxypyrrole-2-carboxamide, (**e**) Calcimycin, (**f**) Nargenicin, (**g**) Phallusialide A and C (R = Cl or Br), and (**h**) Lynamicins A-E: R_1_ = H (A and E) or Cl (B and C); R_2_ = Cl (B, C and D); R_3_ = H (A, B and C) or -COO-CH_3_ (D and E); R_4_ = H (3) or -COO-CH_3_ (A, B, D and E); R_5_ = Cl (A-E); R_6_ = H (A, B, D, and E) or Cl (C).

The chemical structure of several natural compounds includes the pyrrole heterocycle (Figure 2). Some of them are involved in critical biological processes. Examples of such compounds are porphyrin (found in hemoglobin and vitamin B12, metabolized to bile pigments) and chlorophyll [11,16]. Also, a series of alkaloids produced by sponges contain pyrrole and have proven activity against Gram-negative pathogens, e.g., ororoidin, ageliferin, and derivatives (e.g., bromoageliferin and dibromoageliferin) [17,18]. 

The presence of halogenated pyrroles, such as chlorinated and methylated compounds, in bacterial secondary metabolites has been found to exhibit significant biological activities. Notable examples of these compounds include pyoluteorin from *Pseudomonas fluorescens*, pyralomycin from *Nonmuraea spiralis*, pyrrolomycin, marinopyrrole, chlorizidine from Streptomyces, and armeniaspiroles A-C from *Streptomyces armeniacus*. Armeniaspiroles A-C, which possesses a unique chlorinated spiro[4.4]non-8-ene scaffold, has proven potent activity against Gram-positive bacteria, including resistant bacteria, for example, vancomycin-resistant *Enterococcus faecium* (VRE) and methicillin-resistant *Staphylococcus aureus* (MRSA) [19].

### 3.1. Calcimycin 

Calcimycin (A-23187) is known as a calcium ionophore and is derived from *Streptomyces chartreusensis*. Structurally, this compound is a pyrrole polyether-based benzoxazole alkaloid (Figure 2e). Calcimycin is an excellent ionophore selective for divalent cations. Thus, calcimycin can affect proton–cation exchange across biological membranes since it is capable of producing lipophilic complexes with both monovalent cations (Na^+^, K^+^, Li^+^, and Rb^+^) and divalent cations (Mg^2+^, Ca^2+^, and Fe^2+^) [20]. By permitting calcium ions to cross cell membranes that are usually impermeable to Gram-positive bacteria and some fungi, calcimycin exhibits potent efficacy against these pathogens [20,21,22]. There are more natural pyrrole polyether-based benzoxazole alkaloids like cezomycin (a demethylamino analogue of calcimycin), demethyl (C11 position) cezomycin, X-14885A, routiennocin, and AC7230 [20]. 

### 3.2. Lynamycins

Five lynamicins, A–E (Figure 2h), were found to be produced by the unique marine actinomycete NPS12745, which was isolated from a marine sediment sample. These compounds exhibit activity against MRSA and VRE, as well as moderate activity against *Streptococci* and *Haemophilus* pathogens [23]. In addition, lynamicin D’s biological activity was assessed, and it turns out that while it has little impact on cell viability, it can influence pre-mRNA splicing due to its ability to modify the levels of SR protein kinase 1 [24]. 

### 3.3. Marinopyrroles 

Due to their wide range of biological activities, marine pyrrole alkaloids are a significant family of chemical compounds of great interest to chemists. They comprise a considerable group of natural products [25,26]. The reports from recent years on this topic come to support the idea that pyrrole alkaloids are currently of increasing interest. Some marine compounds, especially those with antibacterial activities, have been marketed, and many marine-derived therapeutic compounds are in preclinical or early clinical development [27,28,29,30,31].

Since the discovery of Marinopyrrole A (Figure 2c), which contains an uncommon bispyrrole structure [32], several marinopyrrole derivatives have been considered potential antibiotics against MRSA [32,33]. A bromo derivative of Marinopyrrole A is Marinopyrrole B, obtained from marine Streptomyces sp.; the compound proved effective against MRSA. The marinopyrrole derivatives are the first naturally occurring compounds having a N, C2-linked bispyrrole backbone. These bioactive molecules may help treat infections with antibiotic-resistant bacteria by generating a novel pharmacophore [32].

The mechanism of action is still not elucidated, but Castro-Falcon et al. (2023) advanced the hypothesis that marrinopyrroles act as protonophores. Nevertheless, by creating bigger pores or changing the stability of the peptidoglycan sacculus, they do not appear to compromise the integrity of the cytoplasmic membrane. As per the present model, these acidic substances penetrate the bacterial membrane and move protons within the cell [34].

### 3.4. 1-Methoxypyrrole-2-carboxamide

The natural compound 1-methoxypyrrole-2-carboxamide (Figure 2d) was obtained by Wattanasuepsin W. et al. (2017) from a culture broth of *Streptomyces griseocarneus* SWW368. This pyrrole derivative with a very simple molecular structure presented antibacterial activity against *Staphylococcus aureus, Kocuria rhizophila*, and *Xanthomonas campestris* pv. *Oryzae* (12, 17, and 11 mm inhibition zone diameters at 100 μg/disk) [35].

### 3.5. Nargenicin 

*Nocardia argentinensis* was the source of the pyrrole moiety-containing compound nargenicin (Figure 2f), first discovered in the late 1980s. Its efficacy against Gram-positive bacteria has been demonstrated. The nargenicin antibiotics are members of the macrolide family and have a distinct *cis*-decalin motif joined by an uncommon ether bridge [16,36,37].

### 3.6. Phallusialides A-E

Zhang F. et al. (2019) isolated five novel alkaloids derived from pyrrole, phallusialides A-E (Figure 2g), from *Micromonospora sp*. Two compounds (A and B) demonstrated antibacterial activity against a Gram-positive bacterium (MRSA) and a Gram-negative bacterium *(Escherichia coli)* with MIC values of 32 and 64 μg/mL, respectively. In contrast, the remaining compounds lacked antibacterial activity, providing crucial SAR data for this class of potential antibacterial drugs. Thus, the authors advanced the possibility that halogen substitution (Cl or Br) at the C4 position of pyrrole is closely related to antibacterial activity (in compounds A and B). The extra sugar moieties modulate biological activities; the extra sugars of compounds D and E probably prevent bacterial cell uptake [38].

### 3.7. Phenethylamine Alkaloids 

**Figure 3:**
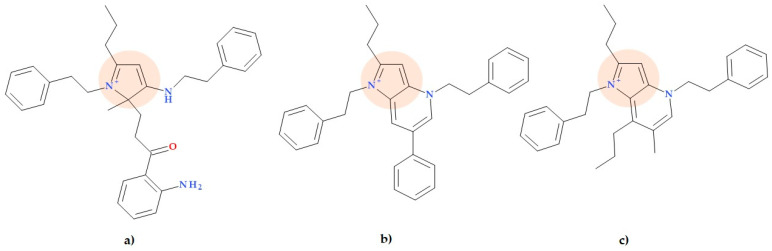
Molecular structure of some phenethylamine alkaloids: (**a**) Dispyrrole, (**b**) Dispyrrolopyridine A, and (**c**) Dispyrrolopyridine B.

Wang L. et al. (2022) evaluated the natural compounds produced by the bacterial genus Tenacibaculum, which have distinct ecological roles in marine environments, and the production of antimicrobial metabolites. Six novel phenethylamine-containing alkaloids were discovered from the medium created by the predatory bacterium. Among these, dispyrrolopyridine A and B (Figure 3) demonstrated intense activity against Gram-positive bacteria: *Staphylococcus aureus, Mycobacterium smegmatis, Bacillus subtilis*, and *Listeria monocytogenes* (MIC values 0.5 to 4 μg/mL). Dispyrrole (Figure 3) showed moderate activity against Gram-positive bacteria. *Escherichia coli* strains lacking an efflux pump were susceptible to dispyrrolopyridine A (MIC values 8 μg/mL) [39].

### 3.8. Pyrrolamides

**Figure 4:**
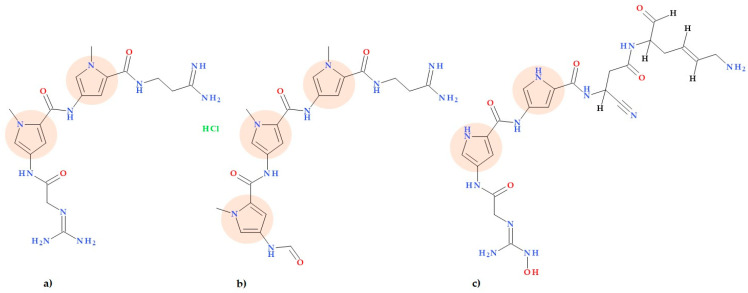
Molecular structure of some natural pyrrolamides: (**a**) Congocidine, (**b**) Distamycin, and (**c**) Pyrronamycin B.

Pyrrolamides are natural products produced by *Streptomyces* and other actinobacteria. These compounds have a poly-pyrrolic structure with one or more pyrrole-2-carboxamide units. Many pyrrolamides, such as congocidine (synanomycin), distamycin (stallimycin), and pyrronamycin B (Figure 4), can bind to specific DNA sequences. This property allows pyrrolamides to exhibit various beneficial biological activities, including antibacterial, antiviral, and antitumor effects [40]. Additionally, potential pyrrolamide antibiotic analogues were isolated by Hao C. et al. (2014) from *Streptomyces netropsis*. A few new analogues of congocidine and distamycin were found and characterized. The authors also proposed a new biosynthesis pathway of pyrrolamides [40]. 

The bacterial cell’s activity is hindered by inhibiting DNA gyrase, a known mechanism of antibacterial activity. AstraZeneca conducted a nuclear magnetic resonance (NMR) screening and a structure-guided design to identify a new class of pyrrolamide compounds. It has been shown that these compounds are potent inhibitors of DNA gyrase; due to this mechanism of action, they have proven antibacterial properties [41].

### 3.9. Pyrrolo-Pyrimidine Derivatives

**Figure 5:**
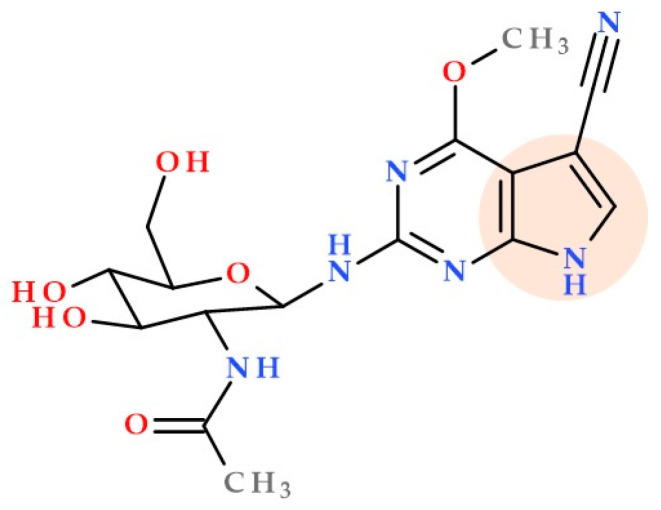
Molecular structure of Huimycin, a natural pyrrole-pyrimidine derivative [42].

Natural pyrrolo-pyrimidines (nucleoside natural compounds) often possess antimicrobial properties (antibacterial, antifungal, antiviral) and anticancer or anti-inflammatory activities. Shuai H. et al. (2020) identified a cluster of biosynthetic genes from the rare actinomycete strain *Kutzneria albida* DSM 43870, producing huimycin. Huimycin (Figure 5) is a new compound member of the pyrrole-pyrimidine family. The 2-amino-6-methoxy-7-cyano-7-deazapurine core has an *N*-acetylglucosamine moiety attached. The huimycin gene cluster was successfully expressed in the heterologous host strain *Streptomyces albus* Del14. Huimycin and dapiramicins A and B are structurally similar, differing only in the presence of a sugar moiety linked to the aglycone. The biosynthesis of huimycin provides a promising platform for developing new pyrrolo-pyrimidines with significant biological activity [42].

### 3.10. Pyrrollomycines

**Figure 6:**
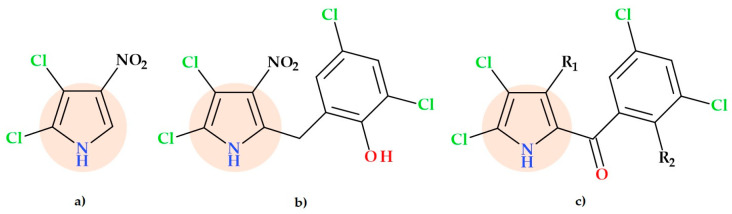
Molecular structure of some natural pyrrolomycines: (**a**) Pyrrolomycin A, (**b**) Pyrrolomycin B, and (**c**) Pyrrolomycin C, D, E, G, H, I and J (R_1_ = -H, -Cl or -NO_2_ and R_2_ = -OH or -OCH_3_).

Pyrolomycins are a class of potent naturally occurring antibiotics that were discovered in the fermentation broth of *Actinosporangium* and *Streptomyces* sp. [43]. Pyrolomycins have nanomolar action against Gram-positive bacteria. Some compounds also exhibit anticancer, neuro-modulatory, immune-modulatory, anthelmintic, and insecticidal activity [44]. Pyrrolomycins are polyhalogenated antibiotics with a stable nitropyrrole nucleus [15,45]. This class of natural antibiotics includes pyrrolomycins (A, B, C, D, E, G, H, I, and J) (Figure 6) and dioxapyrrolomycin [43]. 

Two key structural characteristics of pyrrolomycins are (i) their high degree of halogenation, typically exhibited by chlorine substituents, and (ii) the nitro group’s presence at the pyrrole ring’s position 3 in certain instances [43]. Also, pyrrolomycins are known as potent natural protonophores. Because of their protonophore activity, which enables the selective shuttling of protons across polarized lipid membrane bilayers, pyrrolomycins are potent inhibitors of bacterial membrane potential [45].

### 3.11. PT22 

**Figure 7:**
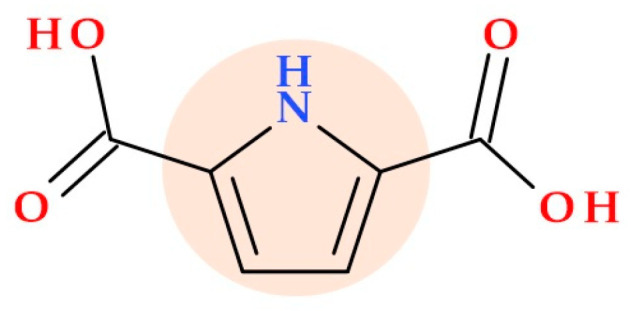
Molecular structure of 1*H*-pyrrole-2,5-dicarboxylic acid (PT22) isolated by Liu J. et al. [46].

A recent study reports a natural dicarboxylic derivative of pyrrole, a quorum-sensing (QS) inhibitor; this compound acts as a new antibiotic accelerator against Gram-negative bacteria when combined with other antibiotics. The compound 1*H*-pyrrole-2,5-dicarboxylic acid (PT22) (Figure 7) isolated from *Perenniporia tephropora* FF2 (an endophytic fungus found in *Areca catechu* L.) by Liu J. et al. (2024) exhibits QS inhibitory activity against *Pseudomonas aeruginosa*. When coadministered with antibiotics (gentamycin or piperacillin), PT22 acts as a promising antibiotic enhancer against *Pseudomonas aeruginosa*. At different concentrations (0.50 mg/mL, 0.75 mg/mL, 1.00 mg/mL), PT22 decreases the production of QS-related virulence factors (such as pyocyanin and rhamnolipid). It inhibits biofilm formation by *Pseudomonas aeruginosa* PAO1 without affecting its growth. Overall, PT22 shows promise as a potent QS inhibitor against *Pseudomonas aeruginosa* antibiotic resistance [46].

### 3.12. Spiroindimycins

**Figure 8:**
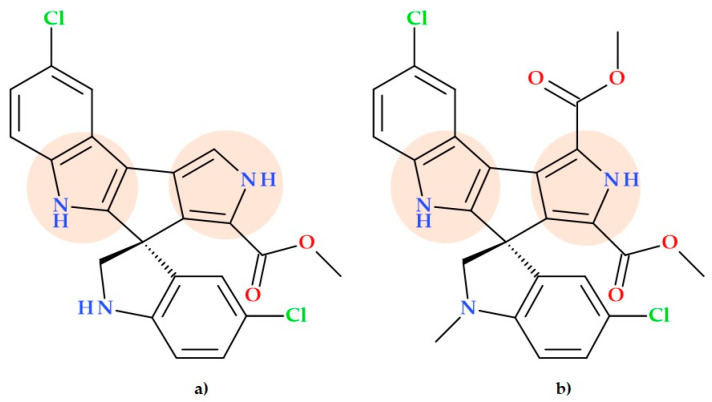
Molecular structure of some natural spiroindomycins: (**a**) Spiroindimycin C, and (**b**) Spiroindimycin D.

Seven drug target enzymes were selected to assess the properties and interactions of spiroindimicins A-D and lynamicins A and D, extracted from *Streptomyces* sp. The pharmacokinetic evaluation (absorption, distribution, metabolization, and elimination—ADME) and toxicity test results suggested that these compounds have the potential to be used as oral drugs. The interaction of the isolated compounds with the target enzymes was found to be satisfactory. Notably, spiroindimycins C and D (Figure 8) and lynamicin D showed inhibition of aromatase P450, cathepsin K, cytochrome P4503A4, histone deacetylase, protein kinase, and topoisomerase II [47].

### 3.13. Spirotetronate Polyketides 

Examination of an extract’s chemical composition from *Actimonadura* sp. and extensive cultivation led to the discovery of six spirotetronates that had not yet been described (Decatromicins C-G and Pyrrolosporin B), as well as six congeners that had been previously identified (BE-45722B-D, Decatromicins A-B, and Pyrrolosporin A). All novel compounds obtained by Ching K.-C. et al. (2022) shared a substantial structural similarity with the spirotetronate-type substances decatromicin and pyrrolosporin, even though the substituents in the aglycone moieties and pyrrole varied. Antibacterial activity was determined; all substances were tested against *Staphylococcus aureus* and *Acinetobacter baumannii*. Among them, BE-45722B, Decatromicin B, and Pyrrolosporin B demonstrated promising antibacterial properties against *Staphylococcus aureus* (MIC90 values from 1 μM to 3 μM) and *Acinetobacter baumannii* (MIC90 values from 12 μM to 36 μM) [48]. 

### 3.14. Streptopyrroles

**Figure 9:**
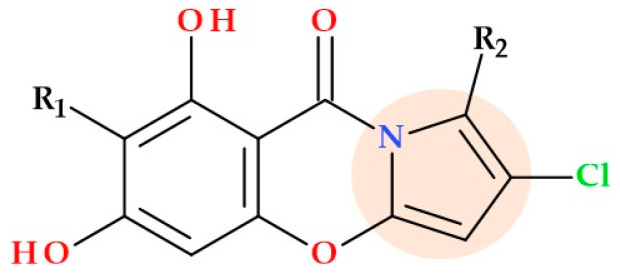
General structure of streptopyrroles isolated from *Streptomyces zhaozhouensis* (R_1_ = n-propyl; n-butyl; iso-butyl; R_2_ = H or Cl) [49].

Heo C.-S. et al. (2023) identified streptopyrroles B and C, two novel alkaloids, and four known analogues from a marine-derived actinomycete (*Streptomyces zhaozhouensis*) (Figure 9). The antibacterial activity of the novel compounds was assessed. These compounds have shown notable antibacterial activity when tested against Gram-positive bacteria such as *Staphylococcus aureus, Bacillus subtilis*, and *Micrococcus luteus*. The MIC values ranged from 0.7 and 2.9 μM, while the positive control, kanamycin, had MIC values under 0.5 μM to 4.1 μM. Overall, monochloride-substituted streptopyrroles exhibit more significant antibacterial activity in SAR studies than dichloride-substituted streptopyrroles because the inclusion of electron-withdrawing groups (halogen atoms) in pyrrole, an aromatic system rich in electrons, could lower its bioactivity. The side chain’s branching and length did not significantly affect antibacterial activity [49].

Since many natural substances exhibit distinctive biological features that could be used for treating infectious diseases, the pharmaceutical industry has always been inspired by nature to develop new antibacterial agents. In the next chapter, we briefly discuss approved drugs from various therapeutic classes that include the heterocycle pyrrole in their molecular structure.

## 4. Drugs Containing Pyrrole Heterocycle

Pyrrole is a significant ring structure in chemistry that exhibits diverse biological activities. Various biologically active compounds are based on pyrrole, where the pyrrole is either a core structure or has various substitutions directly on the heterocycle. Several drugs containing the pyrrole moiety are commercially available, while others are undergoing clinical trials [15]. Pyrrole derivatives have proven potential pharmacological uses as antibacterial, antituberculosis, antiviral, anticancer, antimalarial, antidiabetic, anti-Parkinson’s, and anti-Alzheimer drugs [50]. 

**Figure 10:**
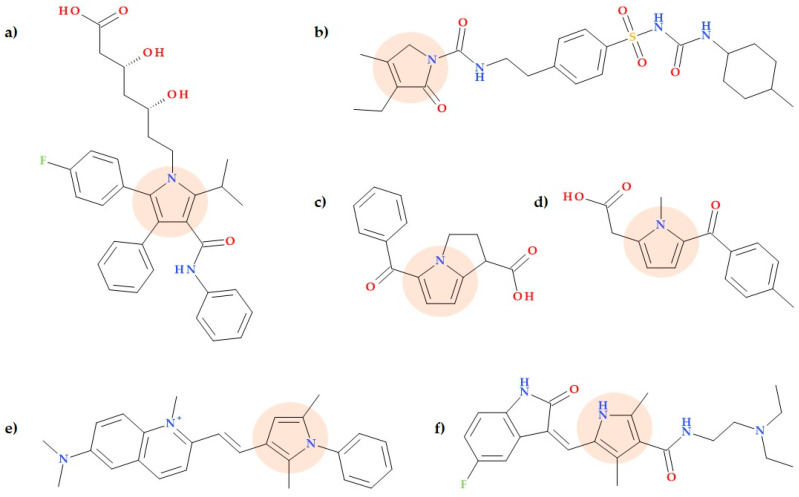
Molecular structure of drugs-based pyrrole heterocycle: (**a**) Atorvastatin, (**b**) Glimepiride, (**c**) Ketorolac, (**d**) Tolmetin, (**e**) Pyrvinium, and (**f**) Sunitinib.

The pyrrole moiety is present in the molecular structure of several approved medicines, such as atorvastatin (a statin representative), glimepiride (a sulfonylurea drug, antidiabetic medication), the nonsteroidal anti-inflammatory drugs (NSAIDs), ketorolac and tolmetin [51,52], pyrvinium (an anthelmintic agent) [53], and sunitinib (a chemotherapeutic drug) (Figure 10) [54].

Other potential drugs are in various clinical phases of study: obatoclax (an anticancer agent in nine phase 2 studies; https://clinicaltrials.gov/study/NCT00682981, accessed on 19 July 2024; https://clinicaltrials.gov/study/NCT00684918, accessed on 19 July 2024), calcimycin (calcium ionophore, in phase 4; https://clinicaltrials.gov/study/NCT04744753, accessed on 19 July 2024), and prodigiosin (different biological activities, in preclinical stage). Also, there are other compounds in various phases of fundamental research, such as ageliferin, aloracetam, elopiprazole, lorpiprazole, isamoltane, and nargenicin [15,50,55]. 

Also, the pyrrole heterocycle can be fused with benzene, cyclohexane, or other heterocycles, for example, in drugs indomethacin (NSAID), fluvastatin (a statin representative), molindone (neuroleptic), sumatriptan (serotonin receptor agonist), tadalafil (phosphodiesterase 5 inhibitor), vemurafenib (a competitive kinase inhibitor), and pexidartinib (selective tyrosine kinase inhibitor) [14,56].

According to our knowledge, no approved antibacterial medicines contain at least one pyrrole heterocycle in the molecular structure. However, we consider it encouraging that several classes of pyrrole derivatives with potential antibacterial activity are outlined and briefly presented in the next chapter.

## 5. New Antibacterial Agents Containing Pyrrole in Their Molecular Structure

Over the past 20 years, there have been remarkable discoveries in the field of pyrrole chemistry [50,57]. These discoveries have indicated that adding heterocyclic moieties to the pyrrole scaffold has increased their bioactivity and produced synergistic effects, which has led to the development of multiple classes of pyrrole analogues [50]. It has been proven that nitrogen heterocyclic derivatives, including pyrrole, exhibit distinctive antibacterial action against different bacterial strains, both Gram-positive and Gram-negative [58]. A promising potential antibacterial action was found when the pyrrole molecule was substituted differently [50]. The antibacterial potential of compounds based on the pyrrole heterocycle with different substituents is extensive. Many studies describing this compound class have been published in recent years [16,50,59].

Without claiming to have identified all the existent studies on the topic in question, the most relevant studies that synthesized new pyrrole derivatives with antibacterial properties, classified by structural groups, are discussed below.

### 5.1. Alkaloids 

Natural alkaloids can be appropriate models for new optimized derivatives with potential antibacterial effects.

**Figure 11:**
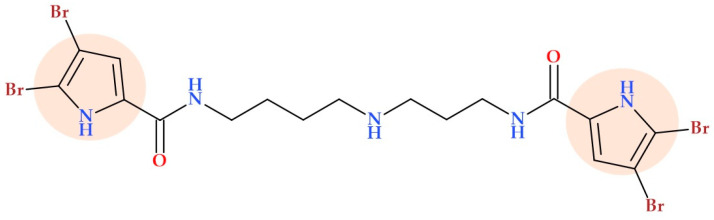
Molecular structure of 4,5-dibromo-*N*-[4-[3-[(4,5-dibromo-1*H*-pyrrole-2-carbonyl)amino]propylamino]butyl]-1*H*-pyrrole-2-carboxamide, a pseudoceratine model compound for designing new analogues with potential antibacterial activity [60].

A total of 65 compounds with different structures were synthesized by Barker D. et al. (2020) based on SAR investigations of 4,5-dibromo-*N*-[4-[3-[(4,5-dibromo-1*H*-pyrrole-2-carbonyl)amino]propylamino]butyl]-1*H*-pyrrole-2-carboxamide (Figure 11), a dibromo pyrrole spermidine alkaloid isolated from the marine sponge *Pseudoceratina purpurea*. After assessing their antibacterial activity, some compounds demonstrated antibacterial activity comparable or superior to that of pseudoceratidin 1 [60].

This family of compounds is generally more effective against Gram-positive bacteria than Gram-negative ones. Additionally, modifying some structural elements enabled the design of a well-defined SAR. Intense antibacterial activity depends on several structural characteristics, including dihalogenation on the pyrrole heterocycle. Also, two pyrrolic units in the structure, either with longer chains or with one or more secondary amines in the subsequent chain, produced more significant antibacterial activity. Analogues with a longer carbon chain may be more successful in penetrating the bacterial cell membrane [60].

### 5.2. Armeniaspirols and Derivatives

**Figure 12:**
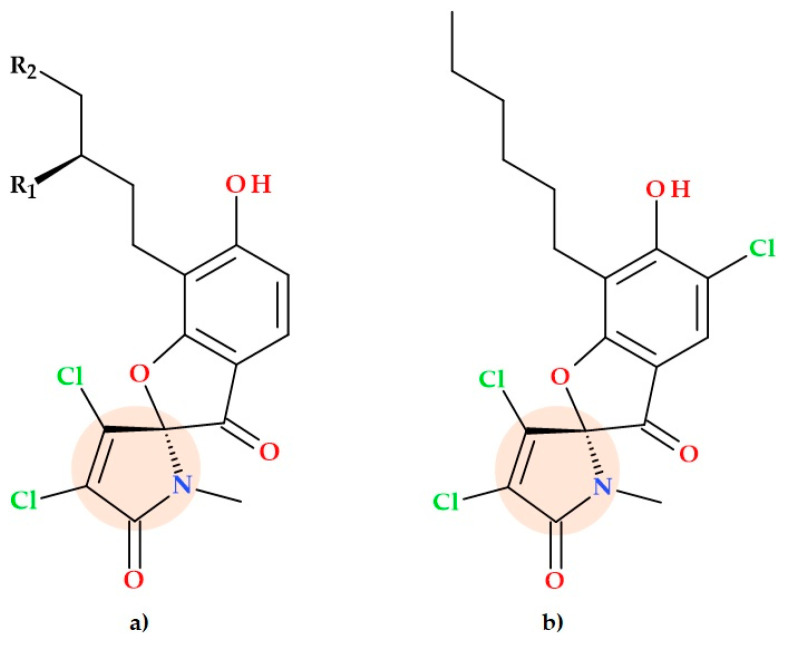
Molecular structure of (**a**) Armeniaspirols A-C (Armeniaspirol A: R_1_ = H, R_2_ = methyl; Armeniaspirol B: R_1_ = methyl, R_2_ = H, Armeniaspirol C: R_1_ = R_2_ = methyl), and (**b**) 5-Chloro-armeniaspirol A.

A spiro[4.4]non-8-ene scaffold distinguishes armeniaspirols, a novel family of natural compounds discovered from *Streptomyces armeniacus*. The 5-chloro analogue of armeniaspirol A was synthesized entirely by Couturier et al. (2012) using a linear six-step technique (Figure 12). The compound 5-chloro-armeniaspirol A presents activity against a variety of Gram-positive bacteria that are resistant to many drugs; the growth inhibition of some pathogens was tested (IC80, μM) and the following values were obtained: 3.7 μM (MRSA), 0.4 μM (penicillin-resistant *Streptococcus pneumoniae*), 8.2 μM (vancomycin-resistant *Enterococcus faecium*) versus standard (vancomycin), 0.4 μM (MRSA), 0.2 μM (penicillin-resistant *Streptococcus pneumoniae*), and >30 μM (vancomycin-resistant *Enterococcus faecium*) [61].

Research on the complex structures of armeniaspirol products allowed Qiao Y. et al. (2019) to generate chemical derivatives through demethylation and dechlorination steps. This aspect led to the discovery of nine new analogues of armeniaspirols, which provided valuable insights into the chlorine atom and methyl group’s role in the pyrrole in antimicrobial activities. *N*-desmethyl armeniaspirols A-C and 9-deschloro armeniaspirols A-C exhibited lower antibacterial activities against *Enterococcus faecium, Enterococcus faecalis*, and *Bacillus subtilis* compared to the chlorinated and methylated compounds. These findings imply that the antibacterial activity of the pyrrole heterocycle depends on the methyl and 9-chloro substituents [19].

### 5.3. 3-Farnesylpyrrole

**Figure 13:**
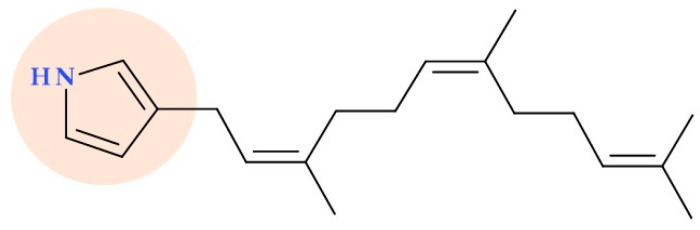
Molecular structure of 3-Farnesylpyrrole [62].

Five new farnesyl-α-nitropyrroles, or nitropyrrolines, with farnesyl groups functionalized at the C4 position, were isolated by Kwon H.C. et al. (2010) by the marine actinomycete strain CNQ-509 (“MAR4” group of marine actinomycetes) abundant in hybrid isoprenoid secondary metabolites. These substances are the first naturally occurring terpenyl-α-nitropyrroles that undergo chemical alteration. 3-Farnesylpyrrole (Figure 13), a synthetic derivative obtained in the structural evaluation process, showed promising antibacterial activity against MRSA (MIC value of 2.8 µg/mL; there are no data about the used standard) [62].

### 5.4. Marinopyrrole Derivatives 

Both marinopyrrole A and its bromo derivative, marinopyrrole B, are members of an uncommon group of bispyrrole natural structures that show promise as antibacterial agents. Also, both natural compounds can be obtained by total synthesis [26,63]. 

**Figure 14:**
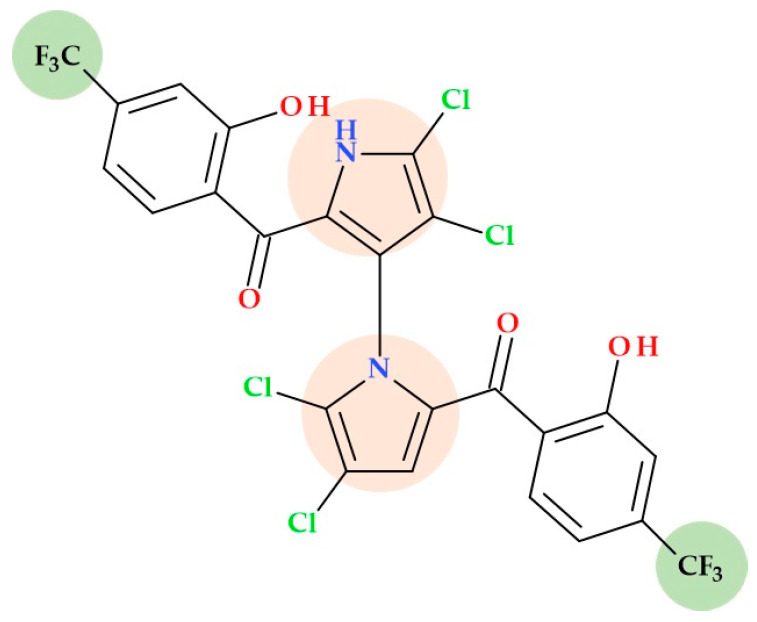
Molecular structure of 4,4’-*para*-trifluoromethyl derivative of Marinopyrrole A synthesised by Cheng C. et al. [33].

Several marinopyrrole derivatives are potential antibiotics against MRSA [32,33]. The SAR study regarding some marinopyrrole derivatives conducted by Cheng C. et al. (2013) highlighted the antibacterial activity of the *para*-trifluoromethyl derivative (Figure 14) of Marinopyrrole A. This derivative showed the highest antibacterial activity because it had *ortho*-hydroxyl groups and electron-withdrawing solid groups (CF_3_) in the *para*-positions. This derivative was proved to be more potent by > 62-fold against methicillin-resistant *Staphylococcus epidermidis* (MRSE) (MIC 8 ng/mL), 8-fold against methicillin-susceptible *Staphylococcus aureus* (MSSA) (MIC 0.125 μg/mL), and 2-fold against MRSA (MIC 0.13–0.255 μg/mL) than standard vancomycin (MIC 0.5–1 μg/mL; 1 μg/mL; 0.5–1 μg/mL) [33].

### 5.5. Pyrrolamides 

**Figure 15:**
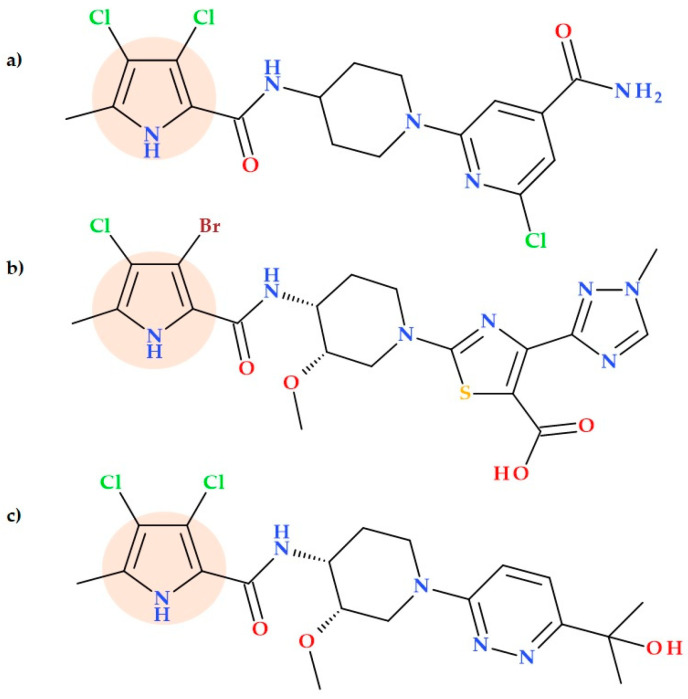
Molecular structure of (**a**) pyrrolamide derivative (2-chloro-6-[4-[(3,4-dichloro-5-methyl-1~{*H*}-pyrrole-2-carbonyl)amino]-1-piperidyl]pyridine-4-carboxamide) obtained by Sherer B.A. et al. [64], (**b**) pyrrolamide derivative (2-[4-[(3-bromo-4-chloro-5-methyl-1*H*-pyrrole-2-carbonyl)amino]-3-methoxy-1-piperidyl]-4-(1-methyl-1,2,4-triazol-3-yl)thiazole-5-carboxylic acid) obtained by Hameed P.S. et al. [65], and (**c**) pyrrolamide derivative (3,4-dichloro-*N*-((3*S*,4*R*)-1-(6-(2-hydroxypropan-2-yl)pyridazin-3-yl)-3-methoxypiperidin-4-yl)-5-methyl-1*H*-pyrrole-2carboxamide) obtained by Zhao X. et al. [66].

Pyrrolamides are potential antibacterial compounds that target DNA gyrase, a vital enzyme to all bacterial species and whose inhibition causes disruption of DNA synthesis and, ultimately, cell death [41,64]. Pyrrolamides with enhanced cellular activity and in vivo efficacy were obtained by substituting pyrrole, piperidine, and heterocycle segments of the molecule to optimize biological activity and other drug-like features. Sherer B.A. et al. (2011) conducted an SAR study of pyrrole substitutions in a pyrrolamide series. Thus, the best in vitro potency (IC50 0.03 μM and MIC 4 μg/mL against *Staphylococcus aureus*) was determined for the 3,4-dichloropyrrole derivate (2-chloro-6-[4-[(3,4-dichloro-5-methyl-1*H*-pyrrole-2-carbonyl)amino]-1-piperidyl]pyridine-4-carboxamide) (Figure 15a). 

In contrast, the weakest activity, with IC50 of 7 μM and MIC > 64 μg/mL against *Staphylococcus aureus*, was determined for the methyl pyrrolamide derivative (2-chloro-6-[4-[(5-methyl-1~{*H*}-pyrrole-2-carbonyl)amino]-1-piperidyl]pyridine-4-carboxamide). The presence of two chlorine atoms (lipophilic electron-withdrawing groups) on the pyrrole heterocycle increases hydrophobic interactions in the adenine pocket and lowers the p*K*a value of the proton on the nitrogen atom of pyrrole. Thus, it can explain the increased activity of the 3,4-dichloropyrrole derivative (Figure 15a), as it results in a stronger hydrogen bond donation to the aspartate residue of the binding domain of GyrB [64]. Using a mouse lung infection model, the same group of researchers proved the effectiveness of a representative pyrrolamide compound against *Streptococcus pneumoniae*. These findings show that pyrrolamides are a new family of DNA gyrase inhibitors that may be used to develop future antibacterial drugs that target various therapeutic purposes [41].

Hameed P.S. et al. (2014) discovered inhibitors from the pyrrolamide class with a general structure of the left-hand-side pyrrole ring, piperidine linker, and right-hand-side thiazole. These inhibitors can effectively kill *Mycobacterium tuberculosis* by blocking the ATPase activity facilitated by the GyrB domain of DNA gyrase. The compound 2-[4-[(3-bromo-4-chloro-5-methyl-1*H*-pyrrole-2-carbonyl)amino]-3-methoxy-1-piperidyl]-4-(3-methyl-3*H*-1,2,4-triazol-5-yl)thiazole-5-carboxylic acid (Figure 15b) was noted in this series as an inhibitor of the activity of DNA gyrase with a 50% inhibitory concentration (IC50) value of < 5 nM. Also, the MIC value of the same compound was 0.03 μg/mL against *Mycobacterium tuberculosis* H37Rv. In the molecular structure of this compound, substituting the pyrrole heterocycle with two halogens can be observed as a beneficial substitution of biological activity. These GyrB inhibitors can potentially become novel antituberculosis agents [65].

Recently, Zhao X. et al. (2023) discovered a new pyrrolamide-type GyrB/ParE inhibitor (3,4-dichloro-*N*-((3*S*,4*R*)-1-(6-(2-hydroxypropan-2-yl)pyridazin-3-yl)-3-methoxypiperidin-4-yl)-5-methyl-1*H*-pyrrole-2carboxamide) (Figure 15c) that has exceptional antibacterial efficacy and druggability against drug-resistant bacterial infections. The MIC value was 0.008 μg/mL (against *Staphylococcus aureus*), and gyrase IC50 was 49 nm/L. Furthermore, this pyrrolamide derivative strongly inhibits *Escherichia coli*, a Gram-negative bacteria (MIC = 1 μg/mL) [66]. The structural optimization of the compound AZD5099, removed from clinical trials, led to the discovery of this particular molecule, a hydroxyisopropyl pyridazine pyrrolamide [66,67]. The hydroxyalkyl moiety on the pyridazine ring could explain the best antibacterial activities and gyrase inhibitory in the series of studied derivatives. Also, this compound has been investigated as a potential preclinical drug for treating Gram-positive bacterial infections that are resistant to drugs [66]. 

### 5.6. Pyrrolyl Benzamide Derivatives 

**Figure 16:**
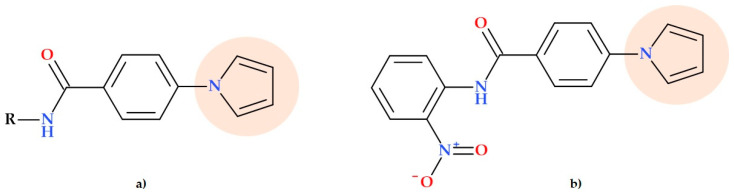
(**a**) General molecular structure of pyrrolyl benzamide derivatives and (**b**) the molecular structure of the compound *N*-(2-nitrophenyl)-4-(1*H*-pyrrol-1-yl) benzamide) designed by Joshi S.D. et al. [68].

Joshi S.D. et al. (2018) synthesized and evaluated new pyrrole benzamide derivatives (Figure 16a) to target the enzyme enoyl-ACP reductase (InhA). InhA is an essential enzyme in the biosynthesis of type II fatty acids from *Mycobacterium tuberculosis*. The researchers evaluated all the pyrrolyl benzamide derivatives as antibiotics against *Mycobacterium tuberculosis* H37Rv and inhibitors of InhA. Among these derivatives, the compound *N*-(2-nitrophenyl)-4-(1*H*-pyrrol-1-yl) benzamide displayed stronger hydrogen bonding interactions with InhA. Compared to all the synthesized derivatives, the in vitro antitubercular results showed that the derivatives with R = 4-ClC_6_H_4_, R = 3-FC_6_H_4_, R = 2,3-ClC_6_H_3_, R = 2-NH_2_C_6_H_4_, and *N*-(2-nitrophenyl)-4-(1*H*-pyrrol-1-yl) benzamide (Figure 16b) are highly active, with an MIC value of 3.125 μg/mL (the used control was isoniazid with an MIC value of 0.25 μg/mL and triclosan with an MIC value of 10 μg/mL). Five compounds were selected for in vitro evaluation of antibacterial activity. These compounds demonstrated more potent activity against *Staphylococcus aureus* comparative to *Escherichia coli*; MIC values were between 3.12 and 12.5 μg/mL (the used control was ciprofloxacin with MIC value of 2 μg/mL for both pathogens). The pyrrole benzamide derivatives served as the lead for creating InhA inhibitors or other effective antibacterial agents [68].

### 5.7. Pyrrole-2-carboxylate and Pyrrole-2-carboxamide Derivatives 

**Figure 17:**
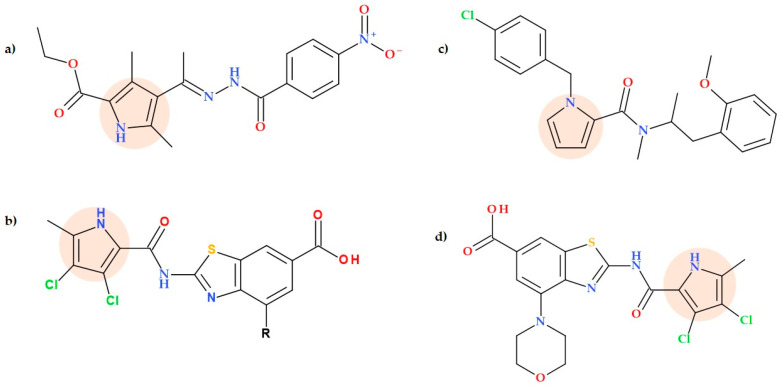
Molecular structure of (**a**) ENBHEDPC pyrrole-carboxylate derivative synthesized by Rawat P. et al. [69], (**b**) the compounds ULD1 and ULD2 obtained by Nyerges A. et al. where ULD1 has R = H and ULD2 has R = -O-CH_2_-C_6_H_5_ [71], (**c**) 1-(4-Chlorobenzyl)-*N*-(1-(2-methoxyphenyl) propane-2-yl)-*N*-methyl-1*H*-pyrrole-2-carboxamide designed by Mane et al. [70], and (**d**) the lead compound (2-(3,4-dichloro-5-methyl-1*H*-pyrrole-2-carboxamido)-4-morpholino-benzo[d]thiazole-6-carboxylic acid) obtained by. Durcik M. et al. [72].

Pyrrole-2-carboxylate was the main pharmacophore in a series of potential antibacterial derivatives. In a study by Rawat P. et al. (2022), four compounds derived from 1*H*-pyrrole-2-carboxylate were synthesized and evaluated for antibacterial activity against *Mycobacterium tuberculosis* H37Rv. Among the compounds, ethyl-4-{[-(1-(2-(4-nitrobenzoyl)hydrazono)ethyl]}-3,5-dimethyl-1*H*-pyrrole-2-carboxylate (ENBHEDPC) (Figure 17a) was found to have an MIC value of 0.7 µg/mL against *Mycobacterium tuberculosis* H37Rv. This MIC value is comparable to the MIC of ethambutol, a standard antimycobacterial drug with an MIC of 0.5 µg/mL. Furthermore, all the obtained derivatives demonstrated activity against *Staphylococcus aureus*, although their MIC values were higher than the standard ceftriaxone [69].

Besides its diverse biological properties, including antibacterial, antibiofilm, and antifungal effects, the pyrrole-2-carboxamide moiety is a pharmacophore in many compounds as a crucial structural component [70]. 

In their study, Nyerges A. et al. (2020) developed unique antimicrobial compounds capable of targeting multiple bacteria. Resistance mutations to these compounds are infrequent, have minimal effects on the susceptibility of the compounds, and significantly decrease bacterial growth. The rational design of balanced multitargeting antibiotics led to two lead compounds of a new chemical class: ULD 1 described as (2-(3,4-dichloro-5-methyl-1*H*-pyrrole-2-carboxamido)benzo[d]thiazole-6-carboxylic acid and ULD2 described as 4-(benzyloxy)-2-(3,4-dichloro-5-methyl-1*H*-pyrrole-2-carboxamido)benzo[d]thiazole-6-carboxylic acid) (Figure 17b) [71].

Mane Y.D. et al. (2017) synthesized some pyrrole-2-carboxamide derivatives and assessed their antibacterial efficacy. Out of all evaluated compounds, seven analogues were the most effective, with MIC values between 1.05 and 12.01 μg/mL against both Gram-positive and Gram-negative bacterial strains:

- 1-(4-Chlorobenzyl)-*N*-(4-methoxybenzyl)-1*H*-pyrrole-2-carboxamide;
- 1-(4-Chlorobenzyl)-*N*-(4-iodobenzyl)-1*H*-pyrrole-2-carboxamide;
- 1-(4-Chlorobenzyl)-*N*-(1-(2-methoxyphenyl)propan-2-yl)-*N*-methyl-1*H*-pyrrole-2-carboxamide;
- 1’-(1-(4-Chlorobenzyl)-1*H*-pyrrole-2-carbonyl)spiro[chroman2,4’-piperidin]-4-one;
- 7-Bromo-1’-(1-(4-chlorobenzyl)-1*H*-pyrrole-2-carbonyl)spiro[chroman-2,4’-piperidin]-4-one;
- *Tert*-butyl 4-(1-(4-chlorobenzyl)-1*H*-pyrrole-2-carboxamido)piperidine-1-carboxylate;
- 1-(4-Chlorobenzyl)-N-cyclohexyl-1*H*-pyrrole-2-carboxamide.

While the compounds 1-(4-chlorobenzyl)-*N*-(4-methoxybenzyl)-1*H*-pyrrole-2-carboxamide and *tert*-butyl 4-(1-(4-chlorobenzyl)-1*H*-pyrrole-2-carboxamido)piperidine-1-carboxylate showed an equal effect to gentamicin against *Pseudomonas aeruginosa*, the compounds 1-(4-chlorobenzyl)-*N*-(1-(2-methoxyphenyl)propan-2-yl)-*N*-methyl-1*H*-pyrrole-2-carboxamide and 1’-(1-(4-chlorobenzyl)-1*H*-pyrrole-2-carbonyl)spiro[chroman2,4’-piperidin]-4-one exhibited the same activity as ciprofloxacin against *Escherichia coli* and *Pseudomonas aeruginosa*, respectively. Out of all the derivatives evaluated, the compound (1-(4-chlorobenzyl)-*N*-(1-(2-methoxyphenyl)propane-2-yl)-*N*-methyl-1*H*-pyrrole-2-carboxamide) (Figure 17c), had significantly higher antibacterial activity against all four bacteria species selected [70].

These novel compounds interact with several amino acids in the target proteins’ ATP-binding sites and inhibit bacterial DNA gyrase and topoisomerase IV complexes nearly equally. The pyrrole-containing compounds are highly potent against many Gram-positive bacteria, including MRSA clinical strains (MIC ≤ 1 μg/mL). The elevated intracellular accumulation of ULD1 and ULD2 in bacterial cells at acidic pH levels is probably the cause of their enhanced bioactivity. Furthermore, the elevated bioactivity of ULD1 and ULD2 in acidic pH environments can be attributed to a higher intracellular accumulation of these molecules within bacterial cells at acidic pH. Therefore, staphylococcal infections could be the first target of therapy with these new potential bacterial agents. ULD1 was demonstrated to have strong effectiveness in vivo in mice with multidrug-resistant staphylococcal skin and thigh infections (topical and systemic administration). These compounds were proven to be effective and non-toxic in testing mice infected with drug-resistant bacteria, suggesting their potential as new treatments for such infections [71].

In another recent study, Durcik M. et al. (2023) [72], based on ULD1 and ULD2 compounds (Figure 17b) [71], designed and synthesized a series of new inhibitors of bacterial DNA gyrase and topoisomerase. These new compounds have demonstrated strong antibacterial properties against various strains of bacteria, including Gram-positive bacteria such as *Enterococcus faecium, Enterococcus faecalis*, and MRSA (with MICs from 0.03125 to 0.25 μg/mL), as well as Gram-negative bacteria like *Acinetobacter baumannii* and *Klebsiella pneumoniae* (with the best compound having an MIC range of 1–4 μg/mL). The lead compound 2-(3,4-dichloro-5-methyl-1*H*-pyrrole-2-carboxamido)-4-morpholino-benzo[d]thiazole-6-carboxylic acid (Figure 17d) has favourable plasma protein binding and solubility, stability, proper metabolism, selectivity for bacterial topoisomerases, and no risk of toxicity. This compound’s crystal structure in complex with *Pseudomonas aeruginosa* has disclosed its manner of binding at the ATP binding site, and further analysis has shown potent antibacterial activity against more than one hundred multidrug-resistant and non-multidrug-resistant strains of *Acinetobacter baumannii*, as well as numerous other Gram-positive and Gram-negative strains. The efficacy of this compound has also been confirmed in a mouse model of vancomycin-mediated *Staphylococcus aureus* thigh infection [72].

### 5.8. Pyrrole-3-carboxaldehyde Derivatives 

**Figure 18:**
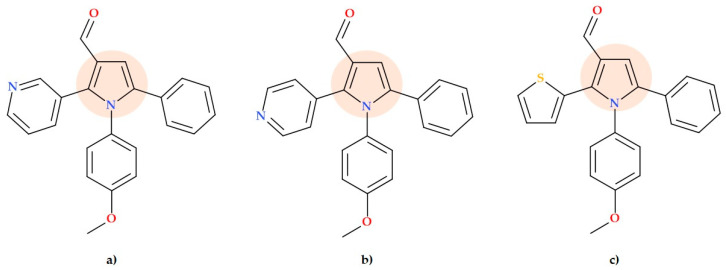
Molecular structure of three substituted pyrrole-3-carboxaldehyde derivatives with activity against *Pseudomonas putida* designed by Mir N.A. et al.: (**a**) 1-(4-Methoxyphenyl)-5-phenyl-2-(pyridin-3-yl)-1*H*-pyrrole-3-carbaldehyde, (**b**) 1-(4-Methoxyphenyl)-5-phenyl-2-(pyridin-4-yl)-1*H*-pyrrole-3-carbaldehyde, and (**c**) 1-(4-Methoxyphenyl)-5-phenyl-2-(thiophen-2-yl)-1*H*-pyrrole-3-carbaldehyde [73].

Pyrrole-3-carboxaldehyde has attracted researchers’ interest as a new pharmacophore. Thus, Mir N.A. et al. (2020) reported the synthesis of highly substituted pyrrole-3-carboxaldehydes. The pyrrole ring was synthesized by the reaction with the aromatic aldehyde and the protected amine. Three selected derivatives displayed significant activity against *Pseudomonas putida* bacteria (MIC value of 16 µg/mL), similar to the used standard (chloramphenicol). The molecular structures of the three compounds are presented below:

- 1-(4-Methoxyphenyl)-5-phenyl-2-(pyridin-3-yl)-1*H*-pyrrole-3-carbaldehyde (Figure 18a);
- 1-(4-Methoxyphenyl)-5-phenyl-2-(pyridin-4-yl)-1*H*-pyrrole-3-carbaldehyde (Figure 18b);
- 1-(4-Methoxyphenyl)-5-phenyl-2-(thiophen-2-yl)-1*H*-pyrrole-3-carbaldehyde (Figure 18c).

The other compounds showed remarkable antibacterial activity in the range of MIC = 32 to 64 μg/mL. Thus, pyrrole-3-carboxaldehyde derivatives substituted at the C2-position with pyridine and thiophene proved substantial antibacterial activity. However, the other two, pyrrole-3-carboxaldehyde with a 4-pyridyl moiety at the C2-position of pyrrole (1-(4-methoxyphenyl)-2-(pyridin-4-yl)-5-p-tolyl-1*H*-pyrrole-3-carbaldehyde and 5-(4-tert-butylphenyl)-1-(4-methoxyphenyl)-2-(pyridin-4-yl)-1*H*-pyrrole-3carbaldehyde), had only modest antibacterial activity [73].

### 5.9. Pyrrole-3-carboxylates 

Several studies have focused on pyrrole-3-carboxylic acid derivatives with antibacterial effects. Massa S. et al. (1990) synthesized and evaluated some 1-arylmethyl-4-aryl-1*H*-pyrrole-3-carboxylic acid analogues as antibacterial agents. A few of them demonstrated antibacterial activity against *Staphylococcus* sp. [74].

**Figure 19:**
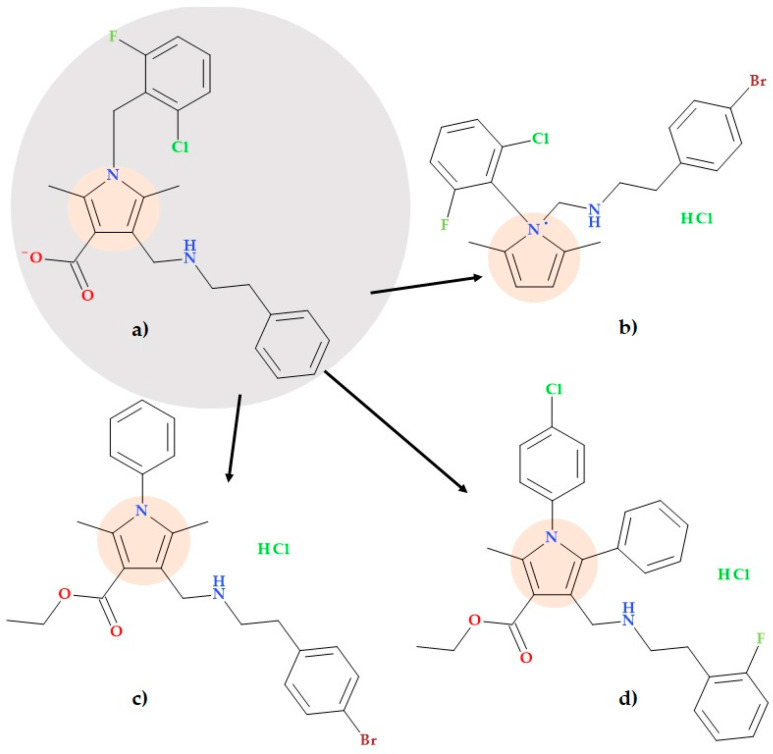
Molecular structure of (**a**) 1-(2-Chloro-6-fluorobenzyl)-2,5-dimethyl-4-((phenethylamino)methyl)-1*H*-pyrrole-3-carboxylate, and its derivatives: (**b**) 2-(4-Bromophenyl)-*N*-((1-(2-chloro-6-fluorophenyl)-2,5-dimethyl-1*H*-pyrrolyl)methyl)ethan-1-amine hydrochloride; (**c**) Ethyl 4-(((4-bromophenethyl)amino)methyl)-2,5-dimethyl-1-phenyl-1*H*-pyrrole-3-carboxylate hydrochloride; (**d**) Ethyl 1-(4-chlorophenyl)-4-(((2-fluorophenethyl)amino)methyl)-2-methyl-5-phenyl-1*H*-pyrrole-3-carboxylate hydrochloride, designed by Liu P. et al. [75].

The development of new antituberculosis agents is crucial in the fight against tuberculosis. High-throughput virtual screening and an in vitro assay were combined by Liu P. et al. (2018) to discover novel inhibitors of *Mycobacterium tuberculosis* caseinolytic proteases. A new pyrrole compound, 1-(2-chloro-6-fluorobenzyl)-2,5-dimethyl-4-((phenethylamino)methyl)-1*H*-pyrrole-3-carboxylate (Figure 19a), showed inhibitory effects against *Mycobacterium tuberculosis* H_37_Ra (MIC of 77 µM). A series of pyrrole derivatives were developed based on this new compound to discover more potent antituberculosis drugs that inhibit ClpP1P2 peptidase in *Mycobacterium tuberculosis*. Three derivatives showed favourable activity with an MIC of 5 µM against *Mycobacterium tuberculosis* H_37_Ra [75], presented below:

- 2-(4-Bromophenyl)-*N*-((1-(2-chloro-6-fluorophenyl)-2,5-dimethyl-1*H*-pyrrolyl)methyl)ethan-1-amine hydrochloride (Figure 19b);
- Ethyl 4-(((4-bromophenethyl)amino)methyl)-2,5-dimethyl-1-phenyl-1*H*-pyrrole-3-carboxylate hydrochloride (Figure 19c);
- Ethyl 1-(4-chlorophenyl)-4-(((2-fluorophenethyl)amino)methyl)-2-methyl-5-phenyl-1*H*-pyrrole-3-carboxylate hydrochloride (Figure 19d).

**Figure 20:**
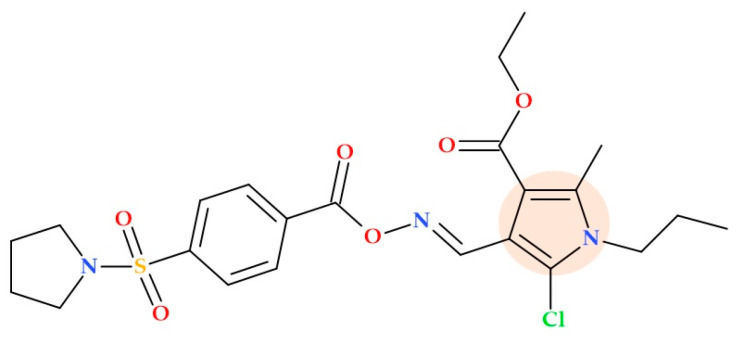
Molecular structure of ethyl 5-chloro-2-methy-1-propyl-4-[({[4-(pyrrolidin-1-ylsulfonyl)benzoyl]oxy}imino)methyl]-1*H*-pyrrole-3-carboxylate synthesized by Grozav A. et al. [76].

Grozav A. et al. (2022) synthesized and tested a series of new O-acyloximes of 4-formylpyrroles derivatives, described as 5-chloro-4-({[alkyl(aryl)oxyimino]methyl})-1*H*-pyrrol-3-carboxylates, for antibacterial activity. These derivatives show antimicrobial activity against *Proteus mirabilis, Proteus vulgaris, Pseudomonas aeruginosa, Klebsiella pneumoniae, Staphylococcus epidermidis, Corynebacterium xerosis*, and *Enterococcus faecalis*. Their MIC values were between 7.81 and 125 μg/mL. Notably, the MIC of compound ethyl 5-chloro-2-methy-1-propyl-4-[({[4-(pyrrolidin-1-ylsulfonyl)benzoyl]oxy}imino)methyl]-1*H*-pyrrole-3-carboxylate) (Figure 20) against *Proteus mirabilis* was 7.81 μg/mL, lower than the corresponding control (Decasan antiseptic is 0.2 mg/mL of decamethoxin; MIC of 15.625 μg/mL) [76].

### 5.10. Pyrrole-3-carbonitriles 

Some studies have focused on obtaining aminopyrrole-3-carbonitrile derivatives with antimicrobial effects. 

**Figure 21:**
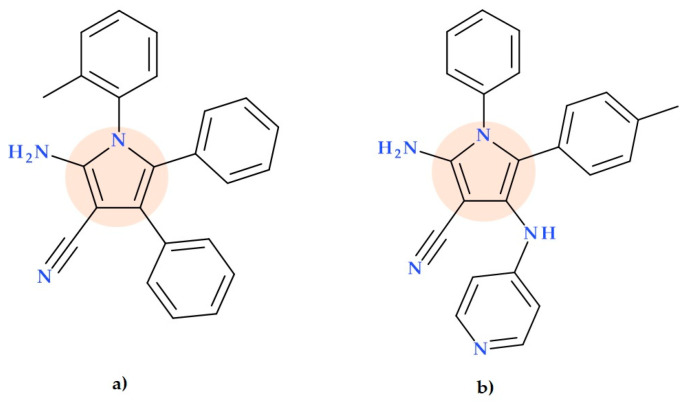
Molecular structure of (**a**) 2-Amino-1-(2-methylphenyl)-4,5-diphenyl-1*H*-pyrrole-3-carbonitrile [77] and (**b**) 2-Amino-1-phenyl-4-(pyridin-4-yl-amino)−5-(*p*-tolyl)−1*H*-pyrrole-3-carbonitrile [78].

Mohammed M.S. et al. (2009) developed four novel 2-aminopyrrole-3-carbonitriles and other pyrrole derivatives. The obtained compounds were tested in vitro for antibacterial activity against Gram-positive and Gram-negative bacteria. The compound 2-amino-1-(2-methylphenyl)-4,5-diphenyl-1*H*-pyrrole-3-carbonitrile (Figure 21a) exhibited strong antibacterial properties compared to amoxicillin against *Escherichia coli* (MIC 32 µg/mL versus 256 µg/mL) [77].

Also, new 2-amino-1,5-diaryl-1*H*-pyrrole-3-carbonitrile derivatives were obtained by Hilmy K.M.H. et al. (2023); these compounds presented potent activities against *Staphylococcus aureus* but were inactive against *Escherichia coli*. Against the *Staphylococcus aureus* strain, the compound 2-amino-1-phenyl-4-(pyridin-4-yl-amino)−5-(p-tolyl)−1*H*-pyrrole-3-carbonitrile (Figure 21b) was distinguished by an MIC value equal to gentamicin (0.008 µM as a positive control). The same compound inhibited DNA gyrase with IC50 of 0.0236 ± 0.45 pM, 1.3 times higher than reference IC50 values of gentamicin (0.0323 ± 0.81 pM) [78].

### 5.11. Pyrrole-Based Chalcone Derivatives

**Figure 22:**
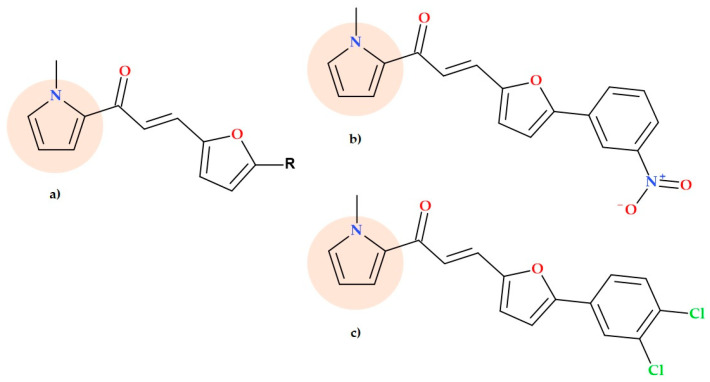
Pyrrole-based chalcone compounds: (**a**) general molecular structure of some pyrrole-based chalcones derivatives, (**b**) 1-(1-Methyl-1*H*-pyrrol-2-yl)-3-(5-(3-nitrophenyl)furan-2-yl)prop-2-en-1-one, and (**c**) 1-(1-Methyl-1*H*-pyrrol-2-yl)-3-(5-(3,4-dichlorophenyl)furan-2-yl)prop-2-en-1-one [79].

Chalcones are a natural target for discovering and developing new anti-infective drugs since numerous studies have demonstrated their antibacterial ability against various bacteria and fungi, including resistant ones [79,80]. By condensation of 2-acetyl-1-methylpyrrole with 5-(aryl)furfural derivatives, pyrrole-based chalcones (Figure 22) were designed and synthesized by Özdemir et al. (2017). The synthesized pyrrole-based chalcones were evaluated in vitro for their antimicrobial effects against some bacterial agents. According to the MIC values, the compound 1-(1-methyl-1*H*-pyrrol-2-yl)-3-(5-(3-nitrophenyl)furan-2-yl)prop-2-en-1-one and the compound 1-(1-methyl-1*H*-pyrrol-2-yl)-3-(5-(3,4-dichlorophenyl)furan-2-yl)prop-2-en-1-one had an antibacterial effect similar to chloramphenicol on *Enterococcus faecalis* (MIC of 100 µg/mL) [79].

### 5.12. Pyrrole Morpholine Derivatives

One of the many biological impacts of morpholin, a multifunctional nucleus, is its antimicrobial properties [81]. For example, in addition to the other crucial structural moieties, the morpholino heterocycle increases the linezolid’s antibacterial activity, which inhibits bacterial protein synthesis (it inhibits the formation of the initiation complex) [82]. Considering the example of linezolid, adding morpholine to pyrrole may be beneficial from the perspective of the antibacterial effect.

**Figure 23:**
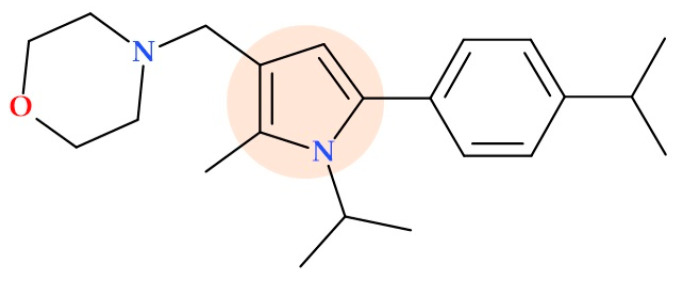
Molecular structure of 4-((1-isopropyl-5-(4-isopropylphenyl)-2-methyl-1*H*-pyrrol-3-yl)methyl)morpholine reported by Poce G. et al. [83].

A series of pyrrole morpholine derivatives with improved physicochemical properties have been reported by Poce G. et al. (2018) as a promising class of antitubercular compounds. The compound 4-((1-isopropyl-5-(4-isopropylphenyl)-2-methyl-1*H*-pyrrol-3-yl)methyl)morpholine (Figure 23) showed excellent activity with an MIC of 0.15 µM against *Mycobacterium tuberculosis* strains susceptible to drugs; this compound demonstrated similar efficacy in a murine model of tuberculosis infection. These pyrrole derivatives would interact with a possible target, namely the *Mycobacterium* membrane protein Large 3 (MmpL3) transporter [83]. 

### 5.13. Pyrrolomycin Derivatives

Numerous synthetic derivatives of pyrrolomycins with antibacterial properties were described by Cascioferro S. et al. (2015) in their review [43].

**Figure 24:**
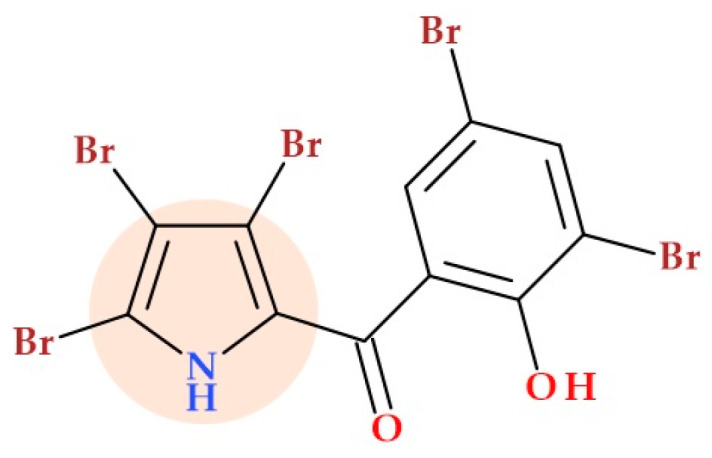
Molecular structure of 3,5-dibromo-2-hydroxy-phenyl)-(3,4,5-tribromo-1*H*-pyrrol-2-yl)methanone obtained by Raimondi M.V. et al. [84].

More recently, Raimondi M.V. et al. (2019) prepared six pyrrolomycins by microwave-assisted synthesis and investigated the mechanism of action of the pyrrolomycins. All compounds showed good inhibitory activity against sortase A (a transpeptidase enzyme responsible for the covalent anchoring of many surface proteins to peptidoglycan from Gram-positive bacteria, thus promoting adhesion and biofilm formation) with IC50 values (130-300 mM) comparable to the hydrochloride of berberine (120 mM). In a docking experiment, the obtained compound 3,5-dibromo-2-hydroxy-phenyl)-(3,4,5-tribromo-1*H*-pyrrol-2-yl)methanone (Figure 24) showed an excellent affinity for sortase A and revealed the most remarkable capacity to prevent *Staphylococcus aureus* from forming biofilms (IC50 of 3.4 nM) [84,85].

Later, Raimondi M.V. et al. (2020) added the nitro groups to the three positions of the pyrrolic nucleus of pyrrolomycin, preserving or boosting their biological activity. The minimal bactericidal concentration (MBC) against *Staphylococcus aureus* or *Pseudomonas aeruginosa* bacteria was improved in comparison to the natural pyrrolomycin C by the nitro substituent’s existence at different pyrrole nucleus positions [44]. 

### 5.14. Pyrrolo-Pyridine Derivatives

The review by Wójcicka A. and Redzicka A. (2021) discusses the biological activity of pyrrolo[3,4-c]pyridines as described in the scientific literature. These compounds have been extensively studied for their potential as analgesic and sedative agents. Also, it was proved that they possess therapeutic potential for treating nervous system disorders and immune system diseases. Additionally, pyrrolo[3,4-c]pyridines have shown antimycobacterial, antiviral, antitumor, and antidiabetic activities. The diverse range of biological activities exhibited by pyrrolo[3,4-c]pyridines highlights their potential as versatile therapeutic agents across multiple disease areas [56]. Some studies that focused on the antibacterial effect of compounds containing a pyrrole moiety fused to a pyridine nucleus are concisely presented further.

**Figure 25:**
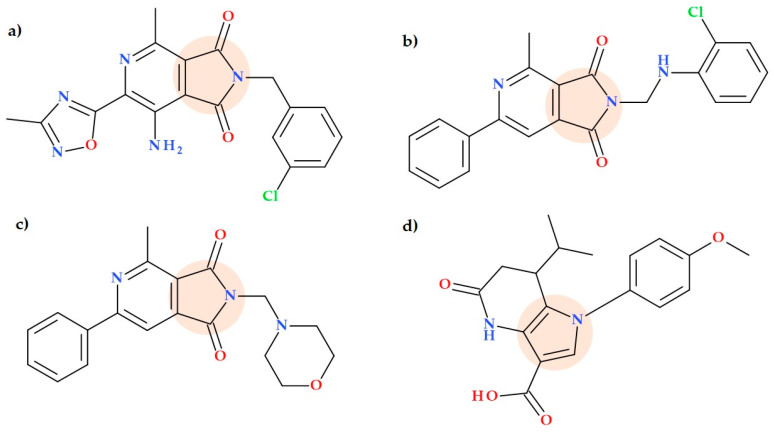
Compounds containing a pyrrole moiety fused to a pyridine nucleus as potential antibacterial agents: (**a**) 7-Amino-2-(3-chlorobenzyl)-4-methyl-6-(3-methyl-1,2,4-oxadiazol-5-yl)-1*H*-pyrrolo[3,4-c]pyridine-1,3-dione [86], (**b**) 2-[(2-Chloranilino)methyl]-4-methyl-6-phenyl-pyrrolo[3,4-c]pyridine-1,3-dione, (**c**) 4-Methyl-2-(morpholinomethyl)-6-phenyl-pyrrolo[3,4-c]pyridine-1,3-dione [87], and (**d**) 7-Isopropyl-1-(4-methoxyphenyl)-5-oxo-6,7-dihydro-4*H*-pyrrolo[3,2-b]pyridine-3-carboxylic acid [88].

7-Amino-2-benzyl-4-methyl-1,3-dioxo-2,3-dihydro-1*H*-pyrrolo[3,4-c]pyridine derivatives were evaluated by van der Westhuyzen R. et al. (2015) for their antimycobacterial activity. The compounds obtained as nitriles and amides presented MIC90 values above 160 µM. However, the esters showed better activity with an MIC90 value below 0.15 µM. The carboxylic acids exhibited moderate activity with an MIC90 value of 3.13 pM, and they had improved solubility and microsomal metabolic stability. It was noted that the pyridine ring’s activity significantly decreased when the methyl group was removed. None of the tested compounds showed toxicity towards VERO cells. The pyrrolo[3,4-c]pyridine derivative with the highest activity, stability, and solubility was 7-amino-2-(3-chlorobenzyl)-4-methyl-6-(3-methyl-1,2,4-oxadiazol-5-yl)-1*H*-pyrrolo[3,4-c]pyridine-1,3-dione (MIC90 = 0.065 μM) (Figure 25a). In vivo, a pharmacokinetic study (conducted on mice) on this compound revealed low plasma exposure, high clearance, and good metabolic stability in liver microsomes [86].

Also, Wojcicka A. et al. (2017) synthesized new pyrrolo[3,4-c]pyridines. Eighteen new compounds were isolated, and their structures were confirmed. Two Mannich bases showed activity against *Staphylococcus aureus*. The compound 2-[(2-chloranilino)methyl]-4-methyl-6-phenyl-pyrrolo[3,4-c]pyridine-1,3-dione (Figure 25b) was able to partially reduce the growth of *Staphylococcus aureus*. Also, the compound 4-methyl-2-(morpholinomethyl)-6-phenyl-pyrrolo[3,4-c]pyridine-1,3-dione (Figure 25c) reduced the growth of *Staphylococcus aureus* [87].

A recent study by Veselov M.S. et al. (2023) discovered a new class of 5-oxo-4*H*-pyrrolo[3,2-b]pyridine derivatives. These compounds showed potent antibacterial activity during a large-scale, high-throughput screening programme. Among the identified compounds, the most active molecule (7-isopropyl-1-(4-methoxyphenyl)-5-oxo-6,7-dihydro-4*H*-pyrrolo[3,2-b]pyridine-3-carboxylic acid) (Figure 25d) had an MIC of 3.35 µg/mL against *Escherichia coli*, indicating its potent antibacterial effect. Additionally, this molecule presented signs of the translational block (as evidenced by a low Katushka2S signal) and no SOS response. Notably, the compound may be a promising candidate for further development as a potential antibacterial agent [88].

### 5.15. Pyrrolyl Pyrimidine Derivatives

**Figure 26:**
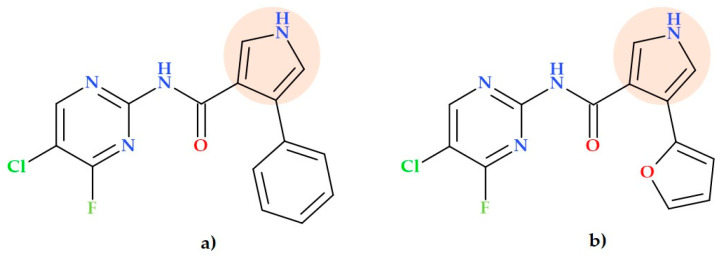
Compounds containing a pyrrole and pyrimidine heterocycles as potential antibacterial agents: (**a**) *N*-(5-Chloro-4-fluoropyrimidin-2-yl)-4-phenyl-1*H*-pyrrole-3-carboxamide and (**b**) *N*-(5-Chloro-4-fluoropyrimidin-2-yl)4-(furan-2-yl)-1*H*-pyrrole-3-carboxamide [89].

Pyrrolyl pyrimidine derivatives with potential antibacterial activity were synthesized by Syamaiah K. et al. (2014). The compound *N*-(5-chloro-4-fluoropyrimidin-2-yl)-4-phenyl-1*H*-pyrrole-3-carboxamide) (Figure 26a) and the compound *N*-(5-chloro-4-fluoropyrimidin-2-yl)4-(furan-2-yl)-1*H*-pyrrole-3-carboxamide) (Figure 26b) demonstrated an antibacterial activity similar to chloramphenicol (the used standard) against *Pseudomonas aeruginosa* (diameter of zone of inhibition was 22 mm and 23 mm versus chloramphenicol, 23 mm; 12.5 µg/well) [89].

### 5.16. Pyrrolo-Pyrimidine Derivatives

According to published reports, fused heterocycles exhibit a variety of biological functions, including antibacterial activity. There is growing interest in pyrrolo-pyrimidines, which have a more varied and potent pharmacological profile than the separate pyrrole and pyrimidine core [90,91].

**Figure 27:**
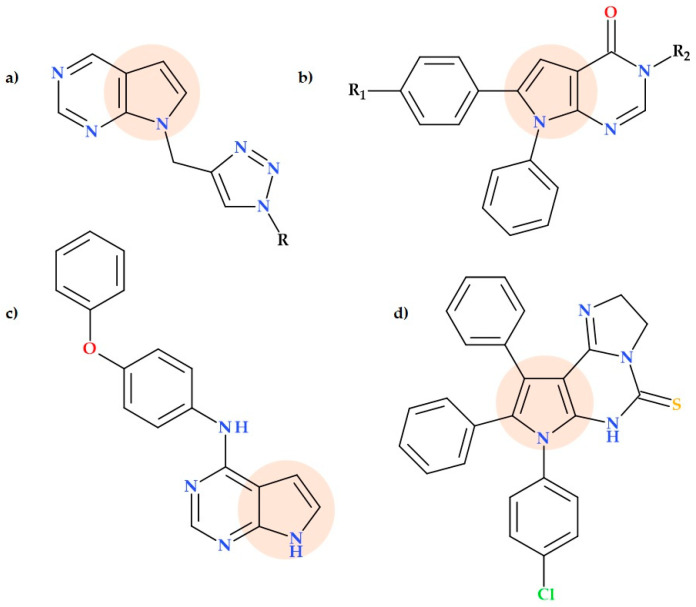
Pyrrole fused with pyrimidine derivatives with potential antibacterial activity: (**a**) pyrrolopyrimidine–triazole hybrids [92], (**b**) pyrrolo[2,3-d]pyrimidine derivatives [93], (**c**) *N*-(4-Phenoxyphenyl)-7*H*-pyrrolo[2,3-d]pyrimidin-4-amine [94], and (**d**) 7–(4-Chlorophenyl)-8,9-diphenyl-6,7-dihydro-2*H*-imidazo[1,2-c]pyrrolo[3,2-e]pyrimidine-5(3*H*)-thione [95].

The synthesis of a series of new 1*H*-pyrrolo[2,3-d]pyrimidine-1,2,3-triazole derivatives was reported by Shiva Raju K. et al. (2019). In vitro, most of these pyrrolopyrimidine–triazole hybrids (Figure 27a) showed promising activity against *Mycobacterium tuberculosis* (MIC of 0.78 µg/mL). Additionally, the SAR analysis demonstrated that the antibacterial activity of the triazole ring was improved by substituting it with heteroaryl compounds that included highly electronegative atoms. According to the study, 1,2,3-triazole is crucial for antitubercular activity [92].

In another study, Hilmy K. et al. (2021) synthesized several new pyrrolo[2,3-d]pyrimidine derivatives (Figure 27b). In comparison to gentamicin (the standard antibiotic), a few of the novel compounds have been found to exhibit promising antibacterial properties. Derivative 6-(4-bromophenyl)-3-[2-(2-hydroxyethoxy)ethyl]-7-phenyl-3,7-dihydro-4*H*-pyrrolo[2,3-d]pyrimidin-4-one was the most efficient against both strains of bacteria in an antibacterial assay against two common bacterial strains (*Escherichia coli* and *Salmonella enteritidis*) with an MIC of 0.5 μg/mL. Both pathogens were favourably susceptible to another obtained derivative, 6-(4-chlorophenyl)-3-(2-hydroxypropyl)-7phenyl-3,7-dihydro-4*H*-pyrrolo[2,3-d]pyrimidin-4one; MIC 3 μg/mL. The activity of two compounds, respectively, 6-(4-bromophenyl)-3-(2,3-dihydroxypropyl)-7-phenyl-3,7-dihydro-4*H*-pyrrolo[2,3-d]pyrimidin-4-one and 2-(4-oxo-6,7-diphenyl-4,7-dihydro-3*H*-pyrrolo[2,3-d]pyrimidin-3-yl)-*N*′-[(2*R*,3*S*,4*R*)-2,3,4,5-tetrahydroxypentylidene]acetohydrazide, was good against *Escherichia coli* and moderate against *Salmonella enteritidis* (MIC 3,5 μg/mL and 8 μg/mL for both compounds) [93].

Lately, the synthesis and SAR of a collection of thirty 7*H*-pyrrolo[2,3-d]pyrimidine derivatives were described by Jesumoroti O.J. et al. (2023). The 7-deazapurine ring’s C-4 position was modified utilizing a variety of aromatic, aryl, and alkyl substituents to create the new compounds. Several SARs have been highlighted for this series. The antitubercular activity is correlated with the halogen’s location on the phenyl ring. Phenyl compounds that have been *ortho*- or *meta*-substituted may easily penetrate bacterial cells and bind their target correctly. The presence of a fluorine atom is unfavourable for antitubercular activity. After comparing compounds with an unsubstituted phenyl ring to those with a 4-morpholino or 4-phenoxyl substituent on the phenyl ring, the former showed rather good to exceptional antitubercular activity; the most effective compound has a 4-phenoxy substituent on the phenyl ring. It was discovered that sixteen derivatives have in vitro activity against the *Mycobacterium tuberculosis* GFP reporter strain; the MIC90 values ranged from 0.488 to 62.5 µM. The compound *N*-(4-phenoxyphenyl)-7*H*-pyrrolo[2,3-d]pyrimidin-4-amine (Figure 27c) was the most promising derivative (MIC90 value of 0.488 µM). Additionally, the ClogP value and molecular weight of each potent molecule in this series are less than 4 and 400, respectively. Consequently, the drug-likeness properties of these derivatives will probably be maintained during lead optimization [94].

Several series of substituted pyrroles and their fused pyrimidine and triazine analogues have been obtained by Abd El-Hameed R.H. et al. (2021). Generally, the obtained compounds inhibited the growth of two Gram-negative bacteria (*Escherichia coli* and *Pseudomonas aeruginosa*) and two Gram-positive bacteria (*Staphylococcus aureus* and *Bacillus subtilis*). Compared to ciprofloxacin, four compounds showed excellent activity against Gram-positive bacteria with higher mean zones of inhibition and lower MIC values. The compound 7–(4-chlorophenyl)-8,9-diphenyl-6,7-dihydro-2*H*-imidazo[1,2-c]pyrrolo[3,2-e]pyrimidine-5(3*H*)-thione (Figure 27d) was the most active, with a mean zone of inhibition value similar to ciprofloxacin (an equivalent MIC against *Pseudomonas aeruginosa*, and lower MIC against *Escherichia coli*) [95]. 

### 5.17. Pyrrole Polymers

The microwave-assisted synthesis of a chitosan-grafted polypyrrole and poly(pyrrole-*N*-(1-naphthyl)ethylenediamine copolymer was reported by Maruthapandi M. et al. (2020). The CFU per mL method was utilized to evaluate the antimicrobial properties of the synthesized polymer materials. Chitosan-grafted polypyrrole completely inhibited the growth of *Escherichia coli*, and the antibacterial effect of poly(pyrrole-*N*-(1-naphthyl)ethylenediamine against *Escherichia coli* was minimal, compared to chitosan-grafted polypyrrole. Similar activities were observed against the *Staphylococcus aureus* pathogen [96].

Also, Elibal F. et al. (2021) synthesized Poly(*N*-methylpyrrole) (PNMPy) samples using the interfacial polymerization method. According to the antibacterial investigation, *Staphylococcus aureus* and *Escherichia coli* bacteria were inhibited by PNMPy samples (inhibition zone of 16 to 21 mm) [97].

### 5.18. Other Pyrrole-Containing Compounds

Numerous other pyrrole-based compounds have been synthesized and tested for antibacterial activity. There is high structural variability among these derivatives. Next, the most relevant studies are briefly presented.

**Figure 28:**
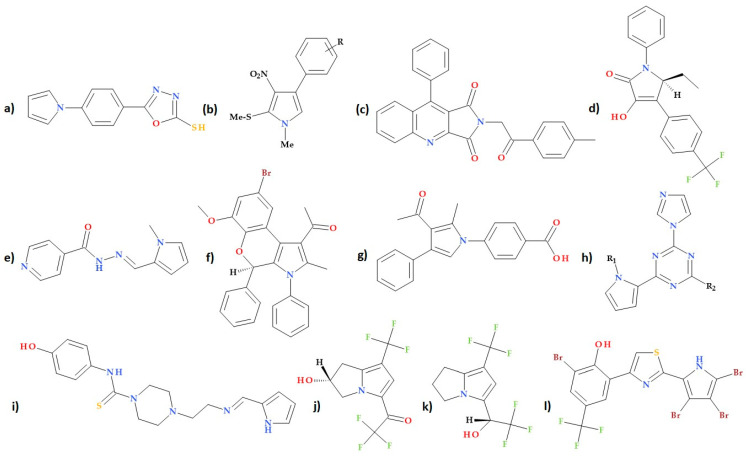
Compounds containing pyrrole in their molecular structure as a potential antibacterials: (**a**) 5-(4-Pyrrol-1-ylphenyl)-1,3,4-oxadiazole-2-thiol [98]; (**b**) Tetra-substituted pyrroles [99]; (**c**) 2-[2-Oxo-2-(p-tolyl)ethyl]-9-phenyl-pyrrolo[3,4-b]quinoline-1,3-dione [100]; (**d**) (*RS*)-5-Ethyl-3-hydroxy-1-phenyl-4-(4-(trifluoromethyl)phenyl)-1,5-dihydro-2*H*-pyrrole-2-one [101]; (**e**) Isonicotinic acid (1-methyl-1*H*-pyrrole-2-yl-methylene)-hydrazide [102]; (**f**) 1-(8-Bromo-6-methoxy-2-methyl-3,4-diphenyl-3,4-dihydrochromeno[3,4-b]pyrrol-1-yl)ethanone [103]; (**g**) (4-(3-Acetyl-2-methyl-4-phenyl-pyrrole-1-yl)benzoic acid) [104]; (**h**) 6-(1-Substituted pyrrole-2-yl)-s-triazine derivatives [105]; (**i**) (*Z*)-4-(2-(((1*H*-Pyrrol-2-yl)methylene)amino)ethyl)-N-(4-hydroxyphenyl)piperazine-1-carbothioamide [106]; (**j**) 2,2,2-Trifluoro-1-[(2R)-2-hydroxy-7-(trifluoromethyl)-2,3-dihydro-1*H*-pyrrolizin-5-yl]ethanone [107]; (**k**) 2,2,2-Trifluoro-1-[7-(trifluoromethyl)-2,3-dihydro-1*H*-pyrrolizin-5-yl]ethanol [107]; (**l**) 2-Bromo-6-(2-(3,4,5-tribromo-1*H*-pyrrol-2-yl)thiazol-4-yl)-4-(trifluoromethyl)phenol [108].

Joshi S.D. et al. (2008) obtained a novel series of pyrrole-containing compounds. All the studied derivatives demonstrated moderate to good bacterial inhibition, according to the analysis of antibacterial screening results, with MIC values between 8 and 500 μg/mL. The compound 5-(4-pyrrol-1-ylphenyl)-1,3,4-oxadiazole-2-thiol (Figure 28a) showed highest antimycobacterial activity (MIC 16 μg/mL) being a promising lead compound [98].

Also, Balachandra B. et al. (2015) developed a particular synthetic method for tetra-substituted pyrroles (Figure 28b). With MIC values ranging from 5.0 to 33.8 μg/mL, all pyrrole derivatives showed potent inhibitory effects against a selection of three Gram-positive bacteria *(Streptococcus pneumoniae, Bacillus subtilis, Bacillus cereus)* and three Gram-negative bacteria *(Salmonella typhi, Escherichia coli, Pseudomonas aeruginosa)* [99].

The study conducted by Hosseyni Largani T. et al. (2017) involved the design and synthesis of a new series of pyrrolo[3,4-b]quinolin-2(3*H*)-yl)benzamides through a molecular hybridization approach. The antibacterial activities of nine compounds from this series were evaluated, and it was found that they exhibited potent inhibition against *Escherichia coli, Staphylococcus aureus*, and *Enterococcus faecalis*. Notably, the compound 2-[2-oxo-2-(*p*-tolyl)ethyl]-9-phenyl-pyrrolo[3,4-b]quinoline-1,3-dione (Figure 28c) demonstrated potent inhibition against *Staphylococcus aureus, Enterococcus faecalis*, and *Escherichia coli*. This compound’s MIC values were 0.25 mg/mL for *Staphylococcus aureus* and *Enterococcus faecalis* and 0.5 mg/mL for *Escherichia coli*. These findings suggest that the synthesized compound has potential as an antibacterial agent, particularly against the tested bacteria [100].

Cusumano A.Q. and Pierce, J.G. (2018), in an SAR study of a series of 3-hydroxy-1,5-dihydro-2*H*-pyrrol-2-one compounds, obtained the lead compound (*RS*)-5-ethyl-3-hydroxy-1-phenyl-4-(4-(trifluoromethyl)phenyl)-1,5-dihydro-2*H*-pyrrol-2-one (Figure 28d), which had MIC values of 8 µg/mL against MRSA, 8–16 µg/mL against linezolid-resistant MRSA, and 2 µg/mL against MRSE [101]. 

A study by Volynets G.P. et al. (2020) focused on the synthesis and antituberculosis activity of new isoniazid-carrying pyrrole analogues. One of the tested compounds, isonicotinic acid (1-methyl-1*H*-pyrrol-2-yl-methylene)-hydrazide (Figure 28e), showed inhibitory activity against the isoniazid-resistant strain SRI 1369 (IC50 value of 0.14 μM). The 1-methyl-1*H*-pyrrol substituent in position R_1_ (and R_2_ = H) was associated with the best antimycobacterial activity (MIC = 0.4 μM). These findings suggest that isonicotinic acid (1-methyl-1*H*-pyrrol-2-yl-methylene)-hydrazide shows promising activity against an isoniazid-resistant strain and warrants further investigation in preclinical studies [102].

A series of 2*H*-chromene-fused pyrrole derivatives were prepared by Baral N. et al. (2020) using a method based on microwave irradiation. The antibacterial activity of the obtained compounds was evaluated against two bacteria (*Staphylococcus aureus* and *Escherichia coli)*. The compound 1-(8-bromo-6-methoxy-2-methyl-3,4-diphenyl-3,4-dihydrochromeno[3,4-b] pyrrol-1-yl)ethanone (Figure 28f) displayed the best antibacterial activity with MIC 20 μg/mL against *Staphylococcus aureus* and *Escherichia coli*, similar to the reference drug, gentamicin. The electron-donating substituent methoxy at the C6 position and electron-withdrawing substituents like bromine at the C8 position of the 2*H*-chromene ring increased the molecule’s potency. A docking study was conducted to find the potential binding orientation of protein DNA gyrase (PDB ID: 3G7E) with the active sites of the chromene-fused pyrrole analogue. According to the data obtained by molecular docking, the compound 15 h had an excellent binding affinity with energy of −8.80 kcal/mol [103].

Also, Akbașlar D.A. et al. (2021) synthesized a series of 1,2,3,4-tetrasubstituted pyrrole derivatives. These compounds exhibited promising antibacterial activity against Gram-positive bacteria (*Staphylococcus aureus* and *Bacillus cereus)*. Their effectiveness was equal to or higher than the standard tetracycline. The presence of electron-withdrawing substituents in the *para* and *meta* positions on the phenyl ring contributed to the enhanced antibacterial potential of these pyrrole derivatives. Among the tested compounds, the compound 4-(3-acetyl-2-methyl-4-phenyl-pyrrol-1-yl)benzoic acid (Figure 28g) containing a carboxyl function at the *para* position had the highest antibacterial activity against *Staphylococcus aureus* and *Bacillus cereus* (inhibition zones of 30 and 19 mm, respectively). However, it is essential to note that none of these derivatives showed any inhibition against the tested Gram-negative bacteria. These findings suggest that the synthesized pyrrole derivatives, particularly the compound with a carboxyl group at the *para* position, may be further explored as potential antibacterial agents against Gram-positive bacteria [104]. 

In a recent study, Green K.D. et al. (2023) described the discovery of 6-(1-substituted pyrrole-2-yl)-s-triazine compounds (Figure 28h), which have shown potent antibacterial activity against *Staphylococcus aureus*, including MRSA strains. These compounds exhibited MICs lower than 1 μM. An imidazole substituent was found to be essential for the antibacterial activity. Additionally, some of the derivatives were also effective against some nontubercular mycobacteria. Notably, the study demonstrated that these molecules are bacteriostatic, inhibiting bacterial growth without killing the bacteria outright. This property is desirable as it allows the immune system to clear the infection effectively. In addition, the compounds were non-toxic to mammalian cells at relevant concentrations. New antibiotics may be designed due to these compounds’ continued development [105].

The study conducted by Velu B. et al. (2023) focused on synthesizing, characterizing, and evaluating the antibacterial activity of a series of pyrrole-coupled carbothioamide derivatives. Among the tested compounds, (*Z*)-4-(2-(((1*H*-pyrrol-2-yl)methylene)amino)ethyl)-*N*-(4-hydroxyphenyl)piperazine-1-carbothioamide (Figure 28i) exhibited intense antibacterial activity against MRSA at a concentration of 18 ± 0.20 µg/mL compared to streptomycin (10 µg/mL). Furthermore, the compound was biocompatible and non-toxic, indicating potential as a drug candidate for combating MRSA infections. The results of this study suggest that this particular pyrrole-coupled carbothioamide derivative may have promising therapeutic applications in the future [106].

Recently, Hodyna D. et al. (2024), based on applying QSAR models, discovered and synthesized six novel bicyclic trifluoromethylated pyrroles. These compounds were tested in vitro against bacteria such as *Staphylococcus aureus, Escherichia coli*, and *Acinetobacter baumannii*. Notably, the compound 2,2,2-trifluoro-1-[(2*R*)-2-hydroxy-7-(trifluoromethyl)-2,3-dihydro-1*H*-pyrrolizin-5-yl]ethanone (Figure 28j) and the compound 2,2,2-trifluoro-1-[7-(trifluoromethyl)-2,3-dihydro-1*H*-pyrrolizin-5-yl]ethanol (Figure 28k) exhibited strong antibacterial effects, particularly against multidrug-resistant strains. The study found that the efficacy of the two compounds is comparable to that of positive control antibiotics against both Gram-positive and Gram-negative bacteria (growth inhibition zones of 13 to 15 mm for 2,2,2-trifluoro-1-[(2*R*)-2-hydroxy-7-(trifluoromethyl)-2,3-dihydro-1*H*-pyrrolizin-5-yl]ethanone and 18 to 22 mm for compound 2,2,2-trifluoro-1-[7-(trifluoromethyl)-2,3-dihydro-1*H*-pyrrolizin-5-yl]ethanol, respectively. In silico studies of the compounds’ molecular mechanism of action by the docking method suggest UDP-N-acetylenolpyruvylglucosamine reductase as a potential target of pyrrole derivatives’ antibacterial effect. A possible target of the antibacterial effect of pyrrole derivatives is UDP-*N*-acetylenolpyruvylglucosamine reductase, according to in silico investigations (molecular docking) of the mechanism of action of the investigated molecules [107].

Also, Zhong Y. et al. (2024) designed and synthesized a series of thiazolyl-halogenated pyrroles or pyrazoles as antibacterial agents and to target biofilms. The compound 2-bromo-6-(2-(3,4,5-tribromo-1*H*-pyrrol-2-yl)thiazol-4-yl)-4-(trifluoromethyl)phenol (Figure 28l) is highlighted due antibacterial activity against some Gram-positive bacteria; in particular, vancomycin-resistant *Enterococcus faecalis* was noted (MIC value equal or under 0.125 μg/mL). Furthermore, at sub-MIC dosages, this new compound dramatically reduced the biofilm formation of *Pseudomonas aeruginosa* and *Staphylococcus aureus* [108]. 

### 5.19. Hybrids and Conjugates

Obtaining hybrids or conjugates has grown in importance in producing various drugs. Molecular hybridization incorporates the pharmacophores of various bioactive substances to create a novel hybrid molecule with complementary activity, multiple targets, and/or counterbalancing adverse effects compared to the original molecules. Numerous efforts to acquire and test hybrids against different bacterial strains over the past few years have been successful [109,110,111].

#### 5.19.1. Curcumin-Based Pyrrole Conjugates

**Figure 29:**
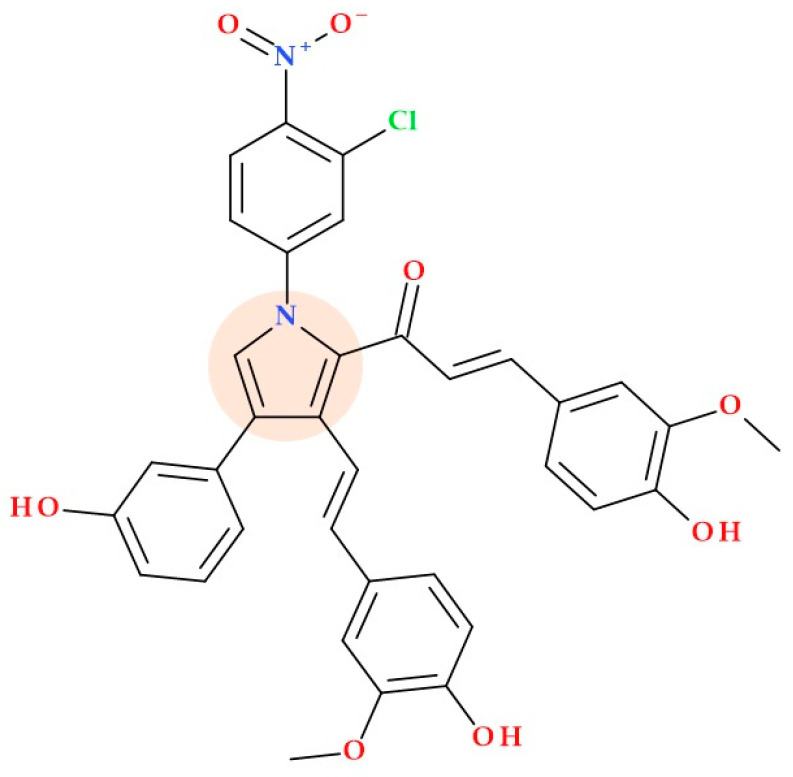
The curcumin-based pyrrole conjugate (*E*)-1-(1-(3-chloro-4-nitrophenyl)-3-((*E*)-4-hydroxy-3-methoxystyryl)-4-(3-hydroxyphenyl)-1*H*-pyrrol-2-yl)-3-(4-hydroxy-3-methoxyphenyl)prop-2-en-1-one, obtained by Gogoi N.G. et al. [112].

Gogoi N.G. et al. (2024) synthesized and studied a set of pyrrole conjugates based on curcumin. These conjugates were tested for their bactericidal efficacy against ESKAP bacterial pathogens, which include *Acinetobacter baumannii, Enterobacter* spp., *Enterococcus* spp., *Klebsiella pneumoniae, Staphylococcus aureus*, and *Pseudomonas aeruginosa*. These conjugates were discovered to have better antibacterial properties than curcumin, particularly against *Staphylococcus aureus* bacteria. For example, the curcumin-based pyrrole conjugate (*E*)-1-(1-(3-chloro-4-nitrophenyl)-3-((*E*)-4-hydroxy-3-methoxystyryl)-4-(3-hydroxyphenyl)-1*H*-pyrrol-2-yl)-3-(4-hydroxy-3-methoxyphenyl)prop-2-en-1-one (Figure 29) presented an MIC value of 16 µg/mL, comparative to the standard levofloxacin (MIC value of 0.25 µg/mL). The binding ways of conjugates to serum albumin proteins and *Staphylococcus aureus* tyrosyl tRNA synthetase have been determined using an in silico molecular docking study. The SAR in this series confirmed that electron-withdrawing groups and multiple phenolic hydroxyl groups on the structure of a curcumin-based pyrrole conjugate increased the antibacterial activity [112].

#### 5.19.2. 4,5-Dibromo-N-phenyl-1H-pyrrole-2-carboxamide Hybrids

**Figure 30:**
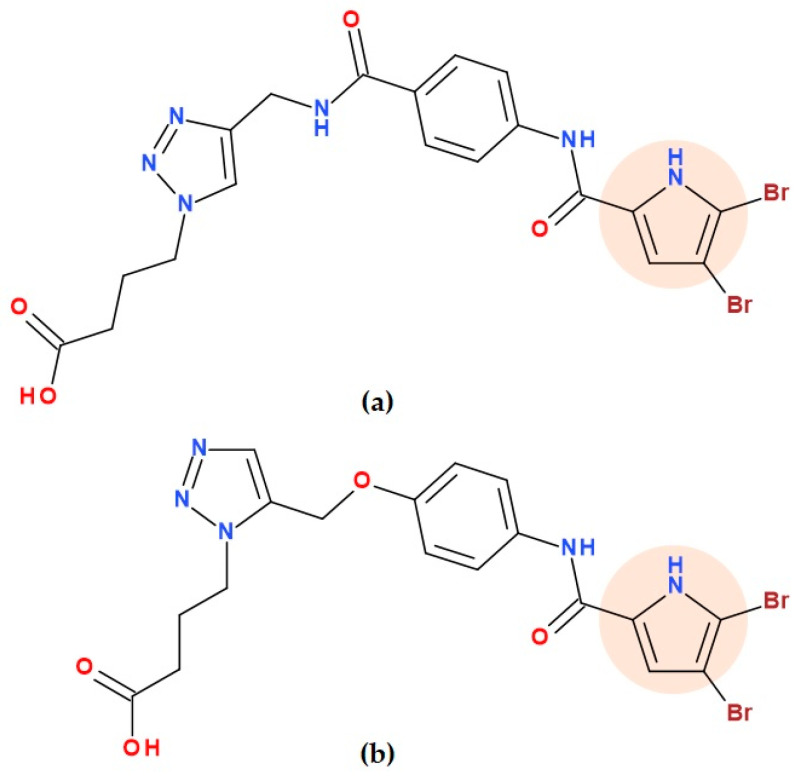
Two hybrids of 4,5-dibromo-*N*-phenyl-1*H*-pyrrole-2-carboxamide with substituted 1,2,3-triazole hybrids had proven activity against *Mycobacterium tuberculosis* H37Rv: (**a**) 4-(4-((4-(4,5-dibromo-1H-pyrrole-2-carboxamido)benzamido)methyl)-1*H*-1,2,3-triazol-1-yl)butanoic acid and (**b**) 4-(5-((4-(4,5-dibromo-1H-pyrrole-2-carboxamido)phenoxy)methyl)-1*H*-1,2,3-triazol-1-yl)butanoic acid [114].

Recently, 1,2,3-triazole and isoxazole rings were successfully hybridized with the antibacterial scaffold 4,5-dibromopyrrole. Merugu S.R. et al. (2024) created and synthesized some new 4,5-dibromo-*N*-phenyl-1*H*-pyrrole-2-carboxamide hybrids using substituted 1,2,3-triazole and isoxazole heterocycles, as potential inhibitors of *Escherichia coli* DNA gyrase. Four hybrids demonstrated good DNA gyrase inhibitory activity. A molecular docking study investigated how the hybrids interact with the target enzyme, DNA gyrase. Gram-negative bacteria were more affected by these hybrid derivatives’ antibacterial activity than Gram-positive ones. Two hybrids were highlighted considering the antibacterial activity: 4-(4-((4-(4,5-dibromo-1*H*-pyrrole-2-carboxamido)benzamido)methyl)-1*H*-1,2,3-triazol-1-yl)butanoic acid (Figure 30a) and 4-(5-((4-(4,5-dibromo-1*H*-pyrrole-2-carboxamido)phenoxy)methyl)-1*H*-1,2,3-triazol-1-yl)butanoic acid (Figure 30b). The antitubercular activity of the two compounds against *Mycobacterium tuberculosis* H37Rv was promising (MIC values of 1.56 µg/mL and 3.125 µg/mL). Good physicochemical characteristics and in vitro safety profile were established for compound 4-(4-((4-(4,5-dibromo-1*H*-pyrrole-2-carboxamido)benzamido)methyl)-1*H*-1,2,3-triazol-1-yl)butanoic acid, which can be optimized and exploited in the future [113].

#### 5.19.3. Hybrids of Sulfonamides Containing the Pyrrole Heterocycle

**Figure 31:**
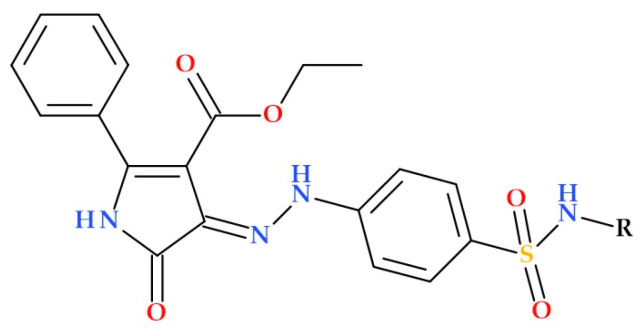
The 5-oxo-2-phenyl-4-(arylsulfamoyl)phenyl)hydrazono)4,5-dihydro-1*H*-pyrrole-3-carboxylate hybrids with remarkable activity against *Salmonella typhimurium* obtained by Gaffer H.E. et al. (R = 2-thiazolyl or 3-pyridinyl) [114].

A recent study aimed to overcome the resistance mechanisms of sulfonamides and identify potential therapeutic approaches through their structural modifications. Gaffer H.E. et al. (2024) developed some 5-oxo-2-phenyl-4-(arylsulfamoyl)phenyl)hydrazono)4,5-dihydro-1*H*-pyrrole-3-carboxylate hybrids to address the bacterial resistance of some sulfonamides. With an inhibition zone diameter of 15 mm and MIC of 19.24 μg/mL, the sulfonamide hybrid ethyl 5-oxo-2-phenyl-4-(2-(4-(*N*-(thiazol-2-yl)sulfamoyl)phenyl)hydrazono)-4,5-dihydro-1*H*-pyrrole-3-carboxylate (Figure 31) demonstrated remarkable antibacterial activity against *Salmonella typhimurium*. Moreover, it substantially inhibited *Escherichia coli*, surpassing the reference sulfamethazole, with an inhibition zone diameter of 19 mm and MIC of 11.31 μg/mL. With an inhibition zone diameter of 16 mm and MIC of 19.24 μg/mL, the hybrid ethyl 5-oxo-2-phenyl-4-(2-(4-(*N*-(pyridin-3-yl)sulfamoyl)phenyl)hydrazono)-4,5-dihydro-1*H*-pyrrole-3-carboxylate also showed intense antibacterial activity against *Salmonella typhimurium* [114].

#### 5.19.4. Pyrrole-1,2,3-triazole Hybrids

**Figure 32:**
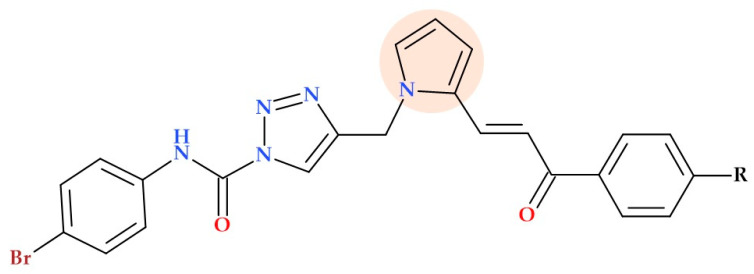
The pyrrole-1,2,3-triazole hybrids obtained by Yadav M. et al. (when R = Br, the hybrid is (*E*)-*N*-(4-bromophenyl)-2-(4-((2-(3-(4-bromophenyl)-3-oxoprop-1-en-1-yl)-1*H*-pyrrol-1-yl)methyl)-1*H*-1,2,3-triazol-1-yl)acetamide; when R = NO_2_, the hybrid is (*E*)-2-(4-((2-(3-(4-bromophenyl)-3-oxoprop-1-en-1-yl)-1*H*-pyrrol-1-yl)methyl)-1*H*-1,2,3-triazol-1-yl)-*N*-(4-nitrophenyl)acetamide) [115].

In a recent study, Yadav M. et al. (2023) reported the synthesis and antibacterial evaluation of pyrrole-1,2,3-triazole hybrids containing chalcone and amide as essential scaffolds. Antimicrobial screening results showed that all synthesized pyrrole-1,2,3-triazoles showed greater efficacy than pyrrole-chalcones against the tested strains. Two compounds, respectively, (*E*)-*N*-(4-bromophenyl)-2-(4-((2-(3-(4-bromophenyl)-3-oxoprop-1-en-1-yl)-1*H*-pyrrol-1-yl)methyl)-1*H*-1,2,3-triazol-1-yl)acetamide and (*E*)-2-(4-((2-(3-(4-bromophenyl)-3-oxoprop-1-en-1-yl)-1*H*-pyrrol-1-yl)methyl)-1*H*-1,2,3-triazol-1-yl)-*N*-(4-nitrophenyl)acetamide (Figure 32), displayed the highest potency toward *Escherichia coli*, similar to standard ciprofloxacin (MIC of 1.56 µg/mL) [115].

#### 5.19.5. Silatran Pyrrole-2-carboxamide Hybrids

**Figure 33:**
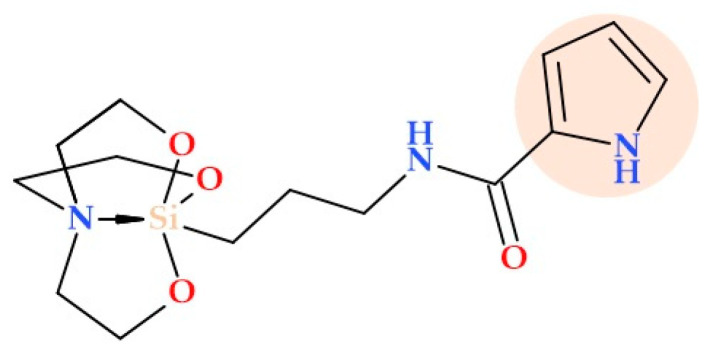
A silatran pyrrole-2-carboxamide hybrid obtained by Adamovich S.N. et al.: *N*-[3-(2,8,9-trioxa-5-aza-1-silabicyclo[3.3.3]undec-1-yl)propyl]-1*H*-pyrrole2-carboxamide [116].

Novel silatrane hybrids with diverse advantageous features, such as anticancer, anti-inflammatory, and antibacterial actions, among others, have been successfully created using molecular hybridization. The study by Adamovich S.N. et al. (2021) focused on synthesizing and evaluating novel silatran pyrrole-2-carboxamide hybrids. These compounds demonstrated low skin permeability, high water solubility, and good gastrointestinal absorption, suggesting their potential for increased bioavailability. Regarding antimicrobial activity, the researchers tested the compounds against Gram-positive bacteria, specifically *Enterococcus durans* and *Bacillus subtilis*. Among the tested compounds, the hybrid *N*-[3-(2,8,9-trioxa-5-aza-1-silabicyclo[3.3.3]undec-1-yl)propyl]-1*H*-pyrrole2-carboxamide (Figure 33) displayed significant activity against both strains (MICs of 3.1 µg/mL and 6.2 µg/mL), compared to the standard gentamicin (MIC of 25 µg/mL) [116].

### 5.20. Metal Complexes

#### Copper Complexes with Pyrrole Hydrazone Derivatives

The study by Joshi S.D. et al. (2016) focused on designing metal complexes using pyrrolyl hydrazones as organic ligands. The biological activity of these complexes was assessed against resistant bacteria species, mainly *Mycobacterium tuberculosis*. The results showed that the copper complexes exhibited significant antitubercular activity, with the highest activity level comparable to that of rifampicin. The MIC of the copper complexes was 0.8 µg/mL. Pyrrolyl hydrazone ligands showed good activity against the tested bacteria (MIC values of 1.6 to 100 µg/mL). The complexes exhibited higher antimicrobial activity than the ligands alone. These findings indicate that the copper ion is essential for increasing the pyrrolyl hydrazones’ biological activity. Overall, this study highlights the potential of pyrrolyl hydrazone metal complexes, specifically copper complexes, as promising candidates for developing new antimicrobial agents, particularly against drug-resistant *Mycobacterium tuberculosis* [117].

## 6. Considerations Regarding the Development of New Pyrrole-Based Antibacterials 

### 6.1. Optimizing the Molecular Structure to Increase the Antibacterial Activity

Some structural modifications led to obtaining promising antibacterial activity against various bacterial species. While some modifications led to antibacterial activity against Gram-positive pathogens, others improved activity against Gram-negative pathogens. Also, some compounds showed potential antimycobacterial activity. The optimization of some compounds led to increased activity in both Gram-negative and Gram-positive bacteria.

#### 6.1.1. Activity Against Gram-Positive Pathogens

Dihalogenation of the pyrrole heterocycle [19,33,60,61], gives the molecule more effects, such as lowering the p*K*a value of the pyrrole nitrogen and increasing the target enzyme’s hydrophobic interactions in its active site [64]. The pyrrole-carboxamide derivatives with substituted pyrrole (3,4-dichloro-5-methyl) (ULD1 and ULD2) are highly potent against multidrug-resistant *Staphylococcus aureus* strains. In addition, these compounds also contain a fragment of benzo[d]thiazole-6-carboxylic, which is favourable for antibacterial activity [71]. Dihalogenated pyrrole associated with a phenyl ring bearing *ortho*-hydroxyl and *para*-trifluorocarbon substituents positively influenced antibacterial activity [33].

Methylated pyrrole from the analogue of armeniaspiroles favoured the activity against *Enterococcus faecium, Enterococcus faecalis*, and *Bacillus subtilis* [19]. Some pyrrole-based chalcones, in which the pyrrole is substituted with a methyl group, demonstrated activity against *Enterococcus faecalis* (similar to chloramphenicol).

The presence of two pyrrolic units is beneficial for antibacterial activity [60]. In addition, the presence of two pyrrolic units associated with chains containing one or more secondary amines or associated with a longer chain was favourable for antibacterial activity [60]. Also, the presence of one pyrrole unit substituted with a long unsaturated chain favoured antibacterial activity [62].

A pyrrole-2-carboxylate pharmacophore was exploited in the molecular structure of four derivatives that can be optimized to become promising antibacterial agents against *Staphylococcus aureus* [69].

Some particular pyrrole-3-carbonitriles derivatives (2-amino-1,5-diaryl-1*H*-pyrrole-3-carbonitrile derivatives) were proven to be potential antibacterials. The compound 2-amino-1-phenyl-4-(pyridin-4-yl-amino)−5-(*p*-tolyl)−1*H*-pyrrole-3-carbonitrile (Figure 21b) was the most active against *Staphylococcus aureus* strains (equal to the gentamicin as a positive control). Antibacterial activity can be obtained even in the absence of halogen substituents. High substitution of pyrrole with phenyl/amino-pyridinyl substituents favours antibacterial activity [78]. Among newly synthesized pyrrolo[3,4-c]pyridines, the compound 2-[(2-chloranilino)methyl]-4-methyl-6-phenylpyrrolo[3,4-c]pyridine-1,3-dione (Figure 25b) and the compound 4-methyl-2-(morpholinomethyl)-6-phenyl-pyrrolo[3,4-c]pyridine-1,3-dione (Figure 25c) reduced the growth of *Staphylococcus aureus* [87].

#### 6.1.2. Activity Against *Mycobacterium tuberculosis*

Pyrrolyl benzamide derivatives have the potential to become antitubercular agents or other efficient antibacterial agents [68]. The 3,5-dimethyl-1*H*-pyrrole is present in the ENBHEDPC compound, a promising antitubercular agent against *Mycobacterium tuberculosis* H37Rv [69]. A pyrrole-2-carboxylate pharmacophore was exploited in the molecular structure of the ENBHEDPC compound, which is also a promising compound with activity against *Mycobacterium tuberculosis* H37Rv [69].

Based on the molecular structure of a highly substituted pyrrole-3-carboxylate derivative, three analogues showed potent activity against *Mycobacterium tuberculosis* H_37_Ra. Two analogues are notable for the structure’s presence of two mono or dihalogenated phenyls (fluorine, chlorine or bromine). All three analogues are characterized by a methyl-ethyl-amino saturated chain substituent to which a phenyl mono halogenate is linked [75].

Adding morpholine to pyrrole was beneficial from the perspective of the antibacterial effect. An illustrative example is the compound 4-((1-isopropyl-5-(4-isopropylphenyl)-2-methyl-1*H*-pyrrol-3-yl)methyl)morpholine (Figure 23), which demonstrated excellent activity against *Mycobacterium tuberculosis* strains susceptible to drugs [83]. A pyrrolo[3,4-c]pyridine derivative, 7-amino-2-(3-chlorobenzyl)-4-methyl-6-(3-methyl-1,2,4-oxadiazol-5-yl)-1*H*-pyrrolo[3,4-c]pyridine-1,3-dione (Figure 25a), presented the highest antimycobacterial activity. The 4-methyl substituent of the pyridine ring favourably influences the antibacterial activity [86].

A series of pyrrolopyrimidine–triazole hybrids (Figure 27a) showed good in vitro activity against *Mycobacterium tuberculosis*. The 1,2,3-triazole is essential for antitubercular activity. Adding heteroaryl compounds with strongly electronegative atoms to the triazole ring improved its antibacterial activity [92].

Metal ions have the potential to increase the antibacterial activity of some ligands. The copper ion plays a crucial role in enhancing the biological activity of the pyrrolyl hydrazones. The complexes exhibited higher antibacterial activity than the ligands alone [117].

#### 6.1.3. Activity Against Gram-Negative Pathogens

Activity against *Escherichia coli* showed pyrrole associated with hydroxyalkyl moiety on the pyridazine ring [66]. Some pyrrole-2-carboxamide derivatives were active against *Pseudomonas aeruginosa* and *Escherichia coli*, equal to gentamicin and ciprofloxacin, respectively [70]. Also, three substituted pyrrole-3-carboxaldehydes displayed significant activity against *Pseudomonas putida* bacterial strains. The pyridine and thiophene heterocycles associated with 1-(4-methoxyphenyl) were favourable substituents for antibacterial activity [73].

The compound ethyl 5-chloro-2-methy-1-propyl-4-[({[4-(pyrrolidin-1-yl-sulfonyl)benzoyl]oxy}imino)methyl]-1*H*-pyrrole-3-carboxylate) showed remarkable activity against the Gram-negative pathogen *Proteus mirabilis*. Notable, the pyrrole is highly substituted: one halogen atom, one methyl, and one propyl substituent, apart from ethyl carboxylate. The other associated structural fragments (e.g., pyrrolidin-1-yl-sulfonyl, benzoyl and oxy-imino) contributed to the increased bacterial activity [76].

Among the new 5-oxo-4*H*-pyrrolo[3,2-b]pyridine derivatives, the compound 7-isopropyl-1-(4-methoxyphenyl)-5-oxo-6,7-dihydro-4*H*-pyrrolo[3,2-b]pyridine-3-carboxylic acid (Figure 25d) was a potent compound against *Escherichia coli* [88]. The probable key moieties are the substituted phenyl with a methoxy group, 7-isopropyl, and 3-carboxyl. Also, this molecule could exist as a zwitterion due to the secondary nitrogen and the carboxylic acid.

The compound *N*-(5-chloro-4-fluoropyrimidin-2-yl)-4-phenyl-1*H*-pyrrole-3-carboxamide and the compound *N*-(5-chloro-4-fluoropyrimidin-2-yl)4-(furan-2-yl)-1*H*-pyrrole-3-carboxamide) (Figure 26) demonstrated activity against *Pseudomonas aeruginosa* similar to chloramphenicol (the used standard) [89]. In these compounds, the presence of a phenyl or furan substituent is favourable for antibacterial activity in combination with a dihalogenated pyrimidine.

#### 6.1.4. Activity Against Gram-Positive and Gram-Negative Pathogens

Seven pyrrole-2-carboxamide derivatives demonstrated intense activity against both Gram-positive and Gram-negative bacterial strains. The structural fragments 4-chlorobenzyl and 2-methoxyphenyl are distinguished in the molecular structure of the compound 1-(4-chlorobenzyl)-*N*-(1-(2-methoxyphenyl)propane-2-yl)-*N*-methyl-1*H*-pyrrole-2-carboxamide [70]. Analogues of ULD1 and ULD2 pyrrole-carboxamide derivatives have demonstrated strong antibacterial properties against some Gram-positive and Gram-negative pathogens. In the structure of the lead compound, the fragment benzo[d]thiazole-6-carboxylic was associated with the morpholine heterocycle, which is favourable for antibacterial activity [72].

The 5-chloro-4-({[alkyl(aryl)oxyimino]methyl})-1*H*-pyrrol-3-carboxylate derivatives show antimicrobial activity against three Gram-positive bacteria (*Staphylococcus epidermidis, Corynebacterium xerosis*, and *Enterococcus faecalis*) and three Gram-negative bacteria (*Klebsiella pneumoniae, Proteus mirabilis, Proteus aeruginosa*, and *Proteus vulgaris*) [76]. 

2-Aminopyrrole-3-carbonitriles derivatives are potential antibacterial compounds. In addition, the high substitution of pyrrole with phenyl substituents favours antibacterial activity, even in the absence of some halogen substituents. Thus, the compound 2-amino-1-(2-methylphenyl)-4,5-diphenyl-1*H*-pyrrole-3-carbonitrile exhibited potent antibacterial properties compared to amoxicillin against *Escherichia coli* [77].

The MBC of some pyrrolomycin derivatives against *Staphylococcus aureus* or *Pseudomonas aeruginosa* pathogen strains was improved by the nitro substituent in various positions of the pyrrole, compared to the natural pyrrolomycin C [44].

Also, tetra-substituted pyrrole derivatives showed potent inhibitory effects against a selection of Gram-positive and Gram-negative bacteria [99].

Numerous structural aspects favourable for antibacterial activity, previously presented, can also be found in the compounds’ molecular structure in Figure 28 (Section 5.18) and the molecular structure of some hybrids and conjugates (Section 5.19). These pyrrolic compounds’ structural variety and antibacterial potential are impressive, from simple pyrrole derivatives to more complex structures, such as pyrrolo[3,4-b]quinolines and isoniazid analogues [100,102]. This variety offers valuable opportunities for optimizing antibacterial activity. In particular, the presence of electron-withdrawing functional groups (e.g., bromo- and trifluoromethyl groups [107,108]) appears to be essential for the improvement of antibacterial activity, as also observed for chromene-fused pyrrole derivatives [103].

These studies highlight a common aspect: compounds containing electron-donating or electron-withdrawing groups strategically placed on the pyrrole heterocycle tend to increase antibacterial activity. For example, in 6-(1-substituted pyrrole-2-yl)-s-triazine compounds [105], an imidazole substituent is crucial for antibacterial potential; this emphasizes the importance of rational drug design, where structural modifications enhance selectivity and efficacy against bacterial targets. Recent observations on the activity of pyrrole-type compounds against drug-resistant bacteria, including MRSA and Gram-negative bacteria, indicate a promising potential for developing new and effective antibacterial agents. The synthesis of hybrid derivatives, such as pyrrolo[3,4-b]quinolines [100], which combine several bioactive cores in a single molecule, could open new horizons in combating antimicrobial resistance. In silico evaluations, such as those that target key bacterial enzymes, could facilitate this progress. Molecular hybridization is a key strategy in the development of new antibacterial compounds. Recent studies on pyrrole hybrids and conjugates have demonstrated a significant improvement in antibacterial activity by introducing favourable structural groups, such as electron-withdrawing and phenolic groups, which enhance interactions with bacterial targets.

Curcumin-based conjugates [112] and carboxamide hybrids [113] showed strong activity against resistant strains, especially *Escherichia coli* and *Staphylococcus aureus*. Also, the structural modifications to sulfonamides [114] and pyrrole-triazole-type compounds [115] have demonstrated increased efficiency in overcoming bacterial resistance mechanisms.

Copper metal complexes with pyrrole derivatives, especially pyrrolyl hydrazones [117], represent a promising direction in developing new antimicrobial agents. Introducing the transition metal, in this case copper, can potentiate the antibacterial activity by facilitating interactions with bacterial cell structures. This mechanism offers opportunities to explore other metals for similar purposes, given the need to develop new therapies against resistant bacteria, especially *Mycobacterium tuberculosis*. All these results underline the importance of optimizing the functional groups and the physicochemical profile of these compounds to obtain new antibacterial agents with increased therapeutic potential.

### 6.2. Possible Mechanisms of Action and Target Enzymes

The compounds with antibacterial potential discussed in this paper target several enzymes or an MmpL3 transporter.

Numerous pyrrole-based compounds interact with the bacterial target enzymes topoisomerases. Pyrrolamides target DNA gyrase [41,64]. The pyrrole-carboxamide derivatives with substituted pyrrole (3,4-dichloro-5-methyl) (ULD1 and ULD2) inhibit bacterial DNA gyrase and topoisomerase IV complexes [71]. Analogues of ULD1 and ULD2 pyrrole-carboxamide derivatives are dual-targeting inhibitors of bacterial DNA gyrase and topoisomerase [72]. Pyrrolamide derivatives are proven to be GyrB inhibitors (antitubercular agents) [65]. Pyrrolamide derivatives, specifically pyrrole associated with a hydroxyalkyl moiety on the pyridazine ring, target GyrB/ParE as an inhibitor [66]. Four 4,5-dibromo-*N*-phenyl-1*H*-pyrrole-2-carboxamide hybrids demonstrated good DNA gyrase inhibitory activity [113]. Thus, inhibiting bacterial topoisomerases could lead to inhibition of bacterial DNA synthesis [118].

Some pyrrolyl benzamide derivatives acted as inhibitors of InhA [68]. Consequently, the synthesis of bacterial mycolic acids and, consequently, the synthesis of the bacterial wall could be inhibited [119].

Six pyrrolomycin derivatives showed good inhibitory activity against the target sortase A (promoting adhesion and biofilm formation) [84]. These pyrrolomycines can potentially reduce biofilm formation in some *Staphylococcus aureus* strains [120].

Some pyrrole derivatives containing morpholine seem to interact with a possible target, MmpL3 transporter (antibacterial effect against *Mycobacterium tuberculosis* strains susceptible to drugs) [83]. MmpL3 is essential for the transport of trehalose monomycolates (precursors of mycolic acid-containing trehalose dimycolates and mycolyl arabinogalactan peptidoglycan); thus, MmpL3 is critical for the mycobacterial cell wall [121].

## 7. Materials and Methods

This review is based on collected references from various databases, including Clarivate Analytics, ScienceDirect, PubMed, and Google Books. The primary keywords used were “pyrrole”, “pyrrole derivatives“, “pyrrole-based compounds“, “substituted pyrroles“, “heterocycles”, “five-membered heterocycles”, and “nitrogen heterocycles” combined with terms like “antibacterials” and “antibiotics”. Additionally, other relevant keywords such as “biological activity”, “drug candidates”, “drug discovery”, “drug design”, “natural antibacterials”, and “natural antibiotics” were considered. Among the obtained references, those concerning pyrrole derivatives with proven antibacterial activity, both natural and synthetic compounds, were selected. Chemical structures were drawn using Biovia Draw 2024 (https://discover.3ds.com/biovia-draw-academic-thank-you, accessed on 26 July 2024) [122] and MarvinSketch 2023 (https://chemaxon.com/marvin, accessed on 19 July 2024) [10] and the IUPAC names of the compounds were obtained from the PubChem database (https://pubchem.ncbi.nlm.nih.gov/, accessed on 26 July 2024) [14].

## 8. Conclusions

Heterocyclic compounds play an essential role in drug design due to their unique structural and electronic properties that allow modulation of therapeutic characteristics, including potency and selectivity. Five-membered heterocycles, especially those containing nitrogen, constitute fundamental structures in many approved antibacterial drugs, demonstrating significant efficacy against infections caused by various pathogens.

As a five-membered heterocycle, pyrrole is distinguished by its versatility in chemical reactions, including electrophilic substitutions, nitration, and sulfonation reactions. This ability to interact with receptors and to be rapidly metabolized gives significant potential in designing new pyrrole-based antibacterial agents. 

Natural compounds based on the pyrrole structure have been used as models for synthesizing new derivatives with antibacterial effects, demonstrating comparable or superior activity to the natural compounds. The diversity of pyrrole derivatives, obtained by various synthesis methods, enables the evaluation of their therapeutic potential. Innovations in shortening the synthetic route led to the rapid availability of these derivatives, facilitating extensive studies on antibacterial activity. Chemical substitutions on the pyrrole nucleus have significantly influenced antibacterial activity, optimizing compounds to target specific bacterial species, including antibiotic-resistant strains. 

Their mechanisms of action include interference with bacterial DNA synthesis, reduction in biofilm formation, and inhibition of mycobacterial cell wall biogenesis, making these compounds promising for developing new antimicrobial agents. The antibacterial activity of these compounds varies with chemical structure and substituents, suggesting that structural changes may influence antibacterial efficacy. Modifying the pyrrolic core (such as dihalogenation or association with other moieties) improved antibacterial activity, especially against drug-resistant bacteria such as *Staphylococcus aureus*. Methyl and amine substituents and long unsaturated chains were favourable for antibacterial activity.

Studies have revealed distinct antibacterial effects of heterocyclic pyrrole derivatives against Gram-positive and Gram-negative bacteria. Compounds including two pyrrolic units or substituents with benzothiazole groups and methyl moieties showed increased activity against Gram-positive bacteria, including *Staphylococcus aureus* and *Enterococcus faecalis* strains. 

Pyrrole derivatives with hydroxyalkyl or benzylsulfonyl moieties have shown activity against important Gram-negative pathogens such as *Escherichia coli* and *Pseudomonas aeruginosa*. SAR studies have shown that the location of the halogen on the phenyl ring influences antimycobacterial activity, and compounds with specific substituents, such as fluorine, are disadvantageous. Derivatives with *ortho*- or *meta*-substituted groups showed an increased ability to penetrate bacterial cells and effective target binding, and the introduction of morpholine into the pyrrolic structures significantly enhanced the antibacterial effect.

Overall, the structural optimization of pyrrole-based compounds, including introducing electron-withdrawing and metallic groups, opens new avenues for developing effective antibacterial agents capable of combating Gram-positive and Gram-negative pathogens, including resistant strains. Recent discoveries highlight the considerable potential of these derivatives for further optimization. Given their favourable drug-likeness characteristics and molecular hybridization, significant progress was achieved in developing new antibacterial agents, demonstrating the future potential to improve antibacterial activity and address antibiotic resistance.

## Figures and Tables

**Table 1:** Nitrogen five-membered heterocycles found in FDA-approved antibacterials (1980–2024) [3].

Nitrogen Five-Member Heterocycle	Antibacterial Compound	FDA Approval Year	Antibacterial Class (Generation)
Pyrrolidine (in a bicycle)	Finafloxacin	2014	Fluoroquinolones
Pyrrolidine	Cefepime	1996	Cephalosporins (4th generation)
	Meropenem	1996	Carbapenems
	Ertapenem	2001	Carbapenems
	Gemifloxacin	2003	Fluoroquinolones
	Ceftobiprole	2009	Cephalosporins (5th generation)
	Doripenem	2014	Carbapenems
	Eravacycline	2018	Tetracyclines
	Gemifloxacin	2018	Fluoroquinolones
	Cefidorocol	2019	Cephalosporins (5th generation)
Pyrrolidine and 1,2,3-Triazole	Cefepime and Enmetazobactam	2024	Cephalosporins (4th generation) and beta-lactamase inhibitor
1,2,3-Triazole	Tazobactam	1992	Beta-lactamase inhibitor
Tetrazole	Cefoperazone	1981	Cephalosporins (3rd generation)
	Cefotiam	1981	Cephalosporins (2nd generation)
	Latamoxef/Moxalactam	1982	Oxacephem cephalosporins (1st generation)
	Cefonicid	1983	Cephalosporins (2nd generation)
	Cefotetan	1987	Cephalosporins (3rd generation)
	Tedizolid	2014	Oxazolidinones
